# Metabolic profiling, antioxidant, and enzyme inhibition potential of *Iris pseudacorus* L. from Egypt and Japan: A comparative study

**DOI:** 10.1038/s41598-023-32224-0

**Published:** 2023-03-30

**Authors:** Suzan M. Yehia, Iriny M. Ayoub, Masato Watanabe, Hari Prasad Devkota, Abdel Nasser B. Singab

**Affiliations:** 1 Department of Pharmacognosy, Faculty of Pharmacy, Ain Shams University, Abbassia, 11566 Cairo Egypt; 2 School of Pharmacy, Kumamoto University, 5-1 Oe-honmachi, Chuo ku, Kumamoto, 862-0973 Japan; 3 Center of Drug Discovery Research and Development, Faculty of Pharmacy, Ain Shams University, Abbassia, 11566 Cairo Egypt

**Keywords:** Secondary metabolism, Mass spectrometry, Metabolomics

## Abstract

Genus *Iris* comprises numerous and diverse phytoconstituents displaying marked biological activities. The rhizomes, and aerial parts of *Iris pseudacorus* L. cultivars from Egypt and Japan were subjected to comparative metabolic profiling using UPLC-ESI-MS/MS. The antioxidant capacity was determined using DPPH assay. In vitro enzyme inhibition potential against *α*-glucosidase, tyrosinase and lipase was evaluated. *In silico* molecular docking was conducted on the active sites of human *α*-glucosidase and human pancreatic lipase. Forty-three compounds were tentatively identified including flavonoids, isoflavonoids, phenolics and xanthones. *I. pseudacorus* rhizomes extracts (IPR-J and IPR-E) exhibited the highest radical scavenging activity with IC_50_ values of 40.89 µg/mL and 97.97 µg/mL, respectively (Trolox IC_50_ value was 14.59 µg/mL). Moreover, IPR-J and IPR-E exhibited promising *α*-glucosidase inhibitory activity displaying IC_50_ values of 18.52 µg/mL, 57.89 µg/mL, respectively being more potent as compared to acarbose with IC_50_ value of 362.088 µg/mL. All extracts exerted significant lipase inhibitory activity exhibiting IC_50_ values of 2.35, 4.81, 2.22 and 0.42 µg/mL, respectively compared to cetilistat with IC_50_ value of 7.47 µg/mL. However, no tyrosinase inhibitory activity was observed for all *I. pseudacorus* extracts up to 500 µg/mL. *In silico* molecular modelling revealed that quercetin, galloyl glucose, and irilin D exhibited the highest fitting scores within the active sites of human *α*-glucosidase and pancreatic lipase. ADMET prediction (absorption, distribution, metabolism, excretion, and toxicity) showed that most of the phytoconstituents exhibited promising pharmacokinetic, pharmacodynamics and tolerable toxicity properties. According to our findings, *I. pseudacorus* might be considered as a valuable source for designing novel phytopharmaceuticals.

## Introduction

The prevalence of some chronic diseases including diabetes mellitus, hypertension, atherosclerosis, and others has rapidly expanded worldwide in the last decade. Therefore, there is a new global orientation for seeking effective and natural constituents for pharmaceutical industries^1^. Oxidative stress can be identified as the disruption in equilibrium between excessive production of oxidants throughout metabolism and presence of low levels of antioxidants within the body leading to cell and organ damage^2^. During the normal process of metabolism, the body releases free radicals at low to moderate concentrations^3^. However, owing to the prevalence of unhealthy diet, lifestyle, and environmental elements such as exposure to pollution and radiation, free radicals are released at high concentrations ^3^. Noteworthy, the occurrence of high levels of free radicals within the body leads to harmful alterations to cell constituents, such as proteins, lipids and DNA. Moreover, several pathological disorders result from this inverse modifications such as diabetes, hypertension, acute respiratory distress syndrome, atherosclerosis, chronic obstructive pulmonary disease, ischemia neurological disorders, and cancer^2^. Therefore, antioxidant agents have a vital role in keeping this balance^3^. There are numerous synthetic antioxidant agents that have been widely used in food and pharmaceutical industries such as butylated hydroxytoluene and butylated hydroxyanisole^4^. These synthetic antioxidants cause several adverse effects including increased risk of cancer, skin allergies and gastrointestinal tract problems^4^. Hence, natural antioxidant agents are generally more preferred nowadays^4^.

The predominance of some chronic illnesses including diabetes mellitus and obesity has rapidly increased implying a global health issue^1^. Different therapeutic approaches are frequently applied including key enzyme inhibitory theory that is considered as one of the most common strategies for controlling these ailments^1^. Several enzymes including *α*-glucosidase, lipase, and tyrosinase are considered as potential targets for lessening symptoms of diabetes mellitus, obesity and skin disorders, respectively^1^. Diabetes mellitus can be defined as a chronic metabolic ailment initiated by abnormal carbohydrate metabolism with a resultant hyperglycemic condition occurring from deficiency in insulin secretion, action, or both^5^. Therefore, the inhibition of *α*-glucosidase enzyme, a member of carbohydrate-digesting enzymes secreted in the small intestine of different organisms, delays glucose absorption and decreases the level of postprandial blood glucose. Hence, diabetes mellitus is controlled^6^. Noteworthy, *α*-glucosidase enzymes have an important role in the breakdown of complex carbohydrates releasing glucose into the small intestine. Glucose is absorbed in the blood circulation leading to increasing the postprandial hyperglycemia^6^. Synthetic *α*-glucosidase inhibitors that have been extensively used in pharmacy for the management of type II diabetic patients, such as acarbose, cause several side effects including flatulence, diarrhea, and abdominal distention^4^. Therefore, there is an increased desire for natural *α*-glucosidase inhibitory agents^6^.

Obesity is a worldwide epidemic chronic disease resulting from accumulation of excessive fat in adipose tissue and leading to complications as hypertension, heart disease, osteoarthritis in joints, hypercholesterolemia, cancer and diabetes mellitus^7^. The cornerstones in the management of obesity are supervised hypocaloric diet, physical exercise, pharmacotherapy and in the most critical cases, bariatric surgery^8^. Related to pharmacotherapy (anti-obesity treatment) is to block pancreatic lipase, a pancreatic enzyme that separates triglycerides into mono acyl glycerol and free fatty acids to ease their absorption^7^. There are many synthetic anti-obesity medications in the market, mainly, orlistat, but it is restricted due to its toxicity to various internal organs including the kidney and liver^7^. Therefore, there is an increased demand for natural-based drugs from plants that contain a substantial amount of lipase inhibitory compounds with minimal side effects.

The enzymatic browning and melanogenesis process that occur in different organisms are carried out by tyrosinase enzyme. Therefore, depigmentation agents are compounds that have the ability for tyrosinase inhibition. These agents are used widely in cosmetics and pharmaceutical formulations. Noteworthy, excessive melanin synthesis leads to various types of skin conditions including cervical poikiloderma, periorbital hyperpigmentation, skin cancer risk and Acanthosis nigricans^9^.

In continuation to our previous work on plants from family Iridaceae^3,10–13^, *Iris pseudacorus* L. was selected in the current study. *Iris pseudacorus* (common name: Yellow flag) is a perennial, monocotyledon, herbaceous and rhizomatous plant with yellow flowers^14^. It is native to Europe, Western Asia and North Africa^15^. Yellow flag grows in a variety of habitats, mostly preferring wetlands, riverbanks, places abundant with water^15^. Thus, its durability makes it a precious plant to consider for the use in wastewater treatment including the uptake of heavy metals, nitrogen and phosphorus wastes or pharmaceutical contaminants as codeine^15^. *I. pseudacorus* has displayed importance in folk medicine^15^. Infusions of the rhizomes were used by traditional Irish and British healers for the treatment of throat inflammations, colds, and toothache^16^. In addition, extracts of the plant were used to treat dandruff, wounds, and as excellent diuretic and tonic^17^. Moreover, the special prepared juice containing *I. pseudacorus* rhizome was used by English healers and administered every hour in syrup of buckthorn to treat dropsy when other medicines failed^18^. Tissues of *I. pseudacorus* accumulate various groups of biologically active substances including phenolic compounds, flavonoids, isoflavonoids, triterpenoids (iridals), organic acids, xanthones, anthocyanins, essential oil and others^14^. Extracts from *I. pseudacorus* could modulate osteoblasts and osteoclasts differentiation and hence, display anti-osteoporotic effects^15^. Besides, *I. pseudacorus* comprises compounds exhibiting estrogenic activity observed in both in vitro and in vivo studies^15^. Essential oils obtained from *I. pseudacorus* rhizomes possess antimicrobial activity against gram negative and positive pathogenic bacteria^15^. Polyphenols such as irilin B and *trans*-3-hydroxy-5,7-dimethoxyflavanone isolated from the roots inhibited the spontaneous colony formation and proliferation of colon carcinoma cells^19^. Iridals *viz.* isoiridogermananl and iridobelamal A isolated from rhizomes exhibited cytotoxic activity against five human tumor cell lines: HL-60, A-549, SMMC-7721, MCF-7 and SW-480^15^. To the best of our knowledge, no reports could be traced regarding the *α*-glucosidase, lipase and tyrosinase inhibitory activity of *I. pseudacorus.*

Therefore, the aim of the current study was to explore the secondary metabolites profile as well as assess the different biological activities of *I. pseudacorus* extracts. Herein, comparative metabolic profiling was achieved using ultra performance liquid chromatography coupled to electrospray ionization mass spectrometry (UPLC-ESI-MS/MS) analysis for the methanol extracts of *I. pseudacorus* rhizomes (IPR) and aerial parts (IPA) from Egypt and Japan. Moreover, the potential antioxidant and enzyme inhibitory activity of *I. pseudacorus* extracts on *α*-glucosidase, lipase and tyrosinase was evaluated in vitro. In addition, *in silico* docking studies were performed to validate the mechanism and binding pattern of the tentatively identified compounds to their targets. Besides ADMET prediction was performed to estimate pharmacokinetics, pharmacodynamics and toxicity properties of these compounds. Hence, the findings of the present study might enhance the knowledge regarding the therapeutic properties of *I. pseudacorus.*

## Results

### Liquid Chromatography Coupled to Mass Spectrometry (LC-MS) Phytochemical Profiles of Egyptian and Japanese *I. pseudacorus* Methanol Extracts

**Figure 1:**
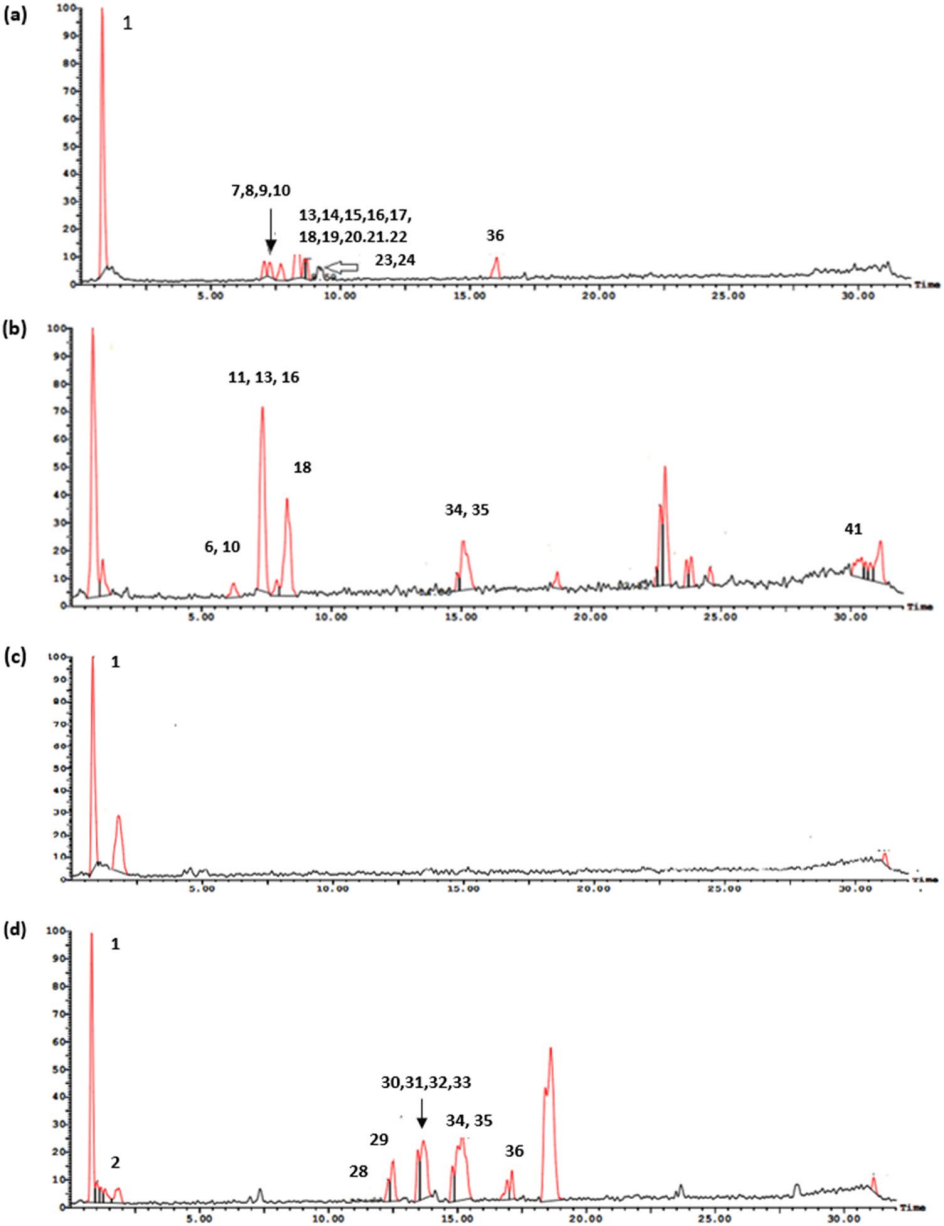
LC/MS base peak chromatograms of IPA-J (**a**), IPA-E (**b**), IPR-J (**c**) and IPR-E (**d**) in negative ionization modes.

**Figure 2:**
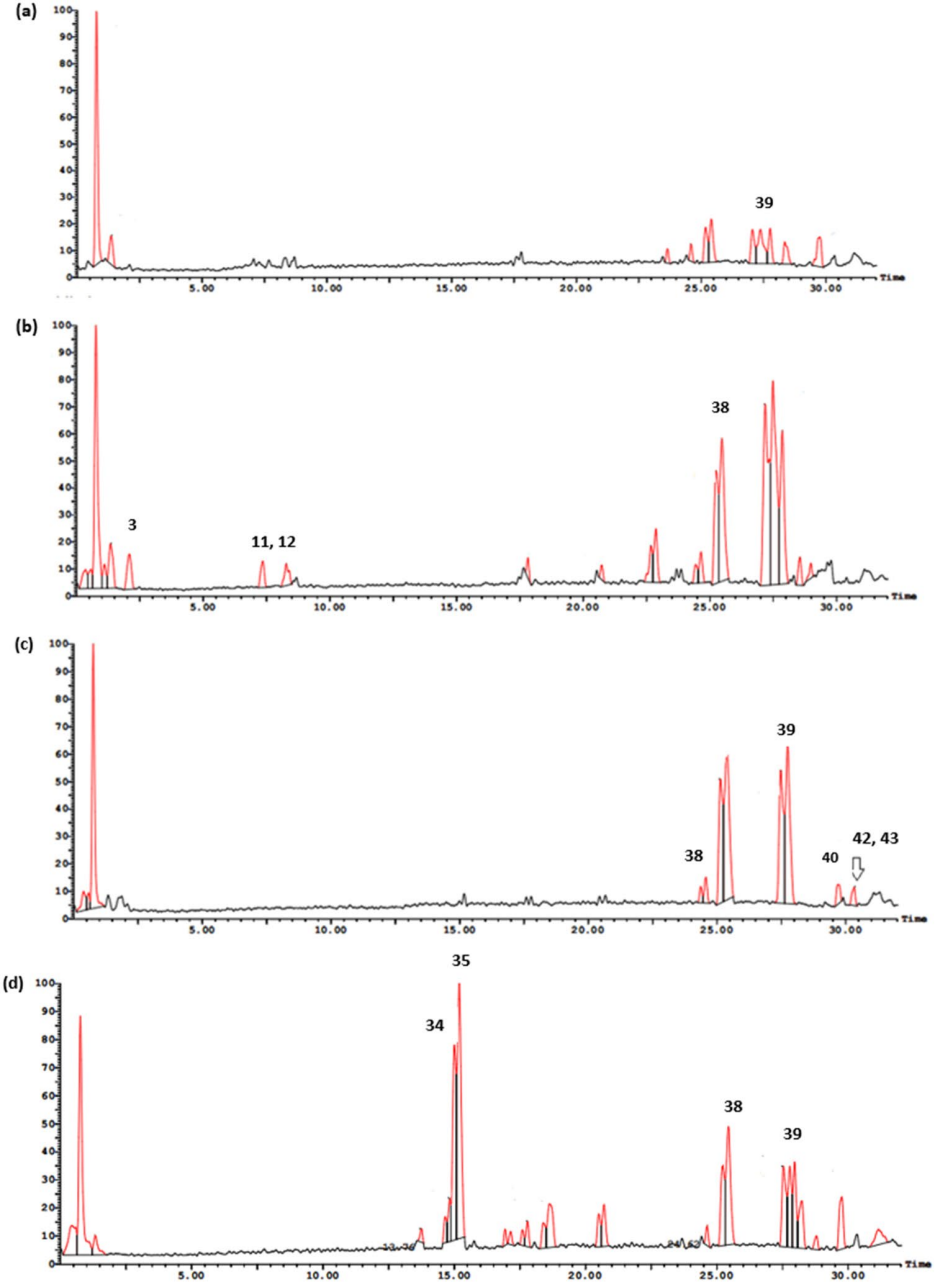
LC/MS base peak chromatograms of IPA-J (**a**), IPA-E (**b**), IPR-J (**c**) and IPR-E (**d**) in positive ionization modes.

**Figure 3:**
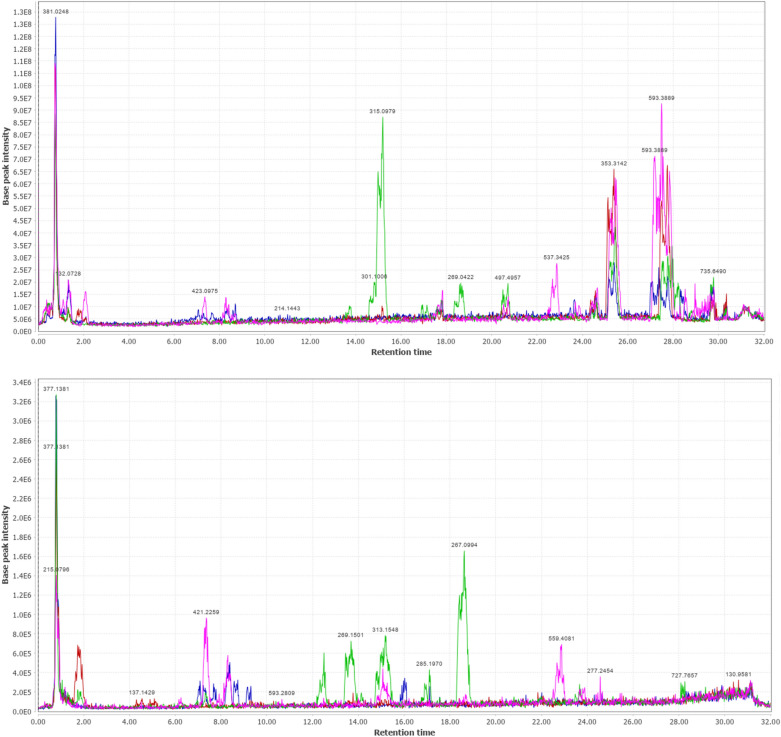
The total ion chromatogram of the aerial parts and rhizomes extracts of *I. pseudacorus* in (**a**) positive, (**b**) negative ionization modes. IPA-J in blue, IPR-J in red, IPA-E in blue, IPR-E in green.

**Figure 4:**
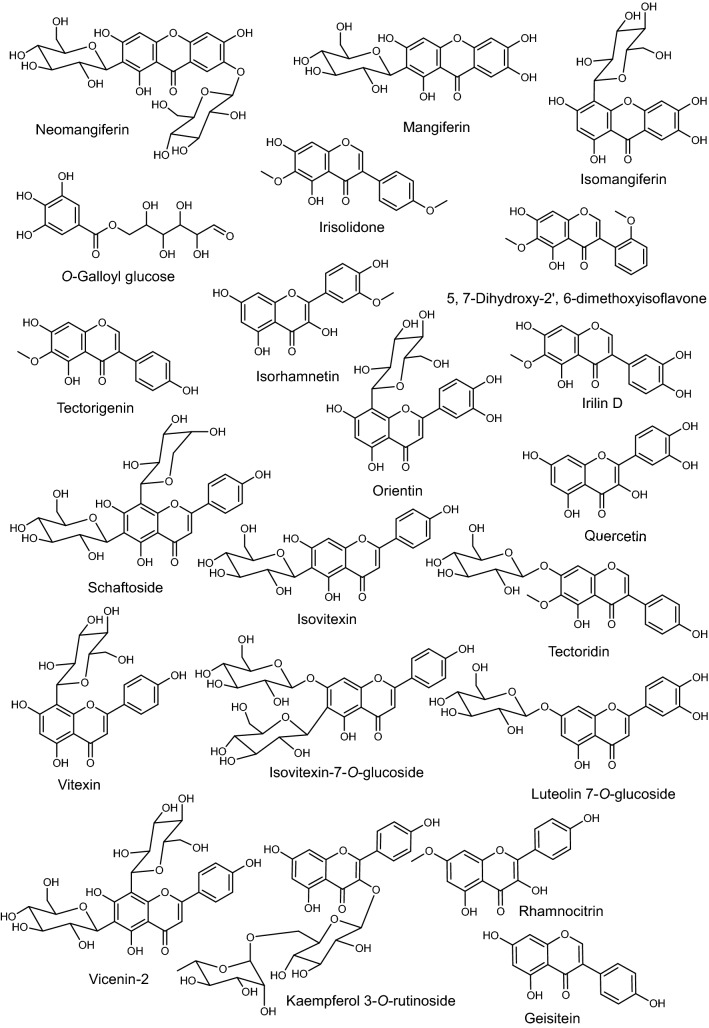
Representative compounds identified in *I. pseudacorus* methanol extracts using UPLC-ESI-MS/MS analysis in both ionization modes.

UPLC-ESI-MS/MS metabolite profiling of rhizomes and aerial parts of *I. pseudacorus* from Egypt and Japan revealed richness in biologically active compounds. The base peak chromatograms of *I. pseudacorus* aerial parts and rhizomes from Japan and Egypt in both negative and positive ionization modes are displayed in Figs. 1, 2 and 3. The tentatively identified compounds are summarized in Table 1 and illustrated in Fig. 4. The number of identified compounds in each class and their distribution are displayed in Table 2. Forty-three compounds were tentatively identified based on comparison of mass spectral data with literature database^20–24^. Compounds belonged to various classes including phenolics, flavonoids, isoflavonoids and xanthones.

**Table 1:** Secondary metabolites identified by UPLC-ESI-MS/MS analysis of *I. pseudacorus extracts* in both negative and positive ionization modes.

No.	Rt (min)	[M-H]^-^	[M+H]^+^	MS^*n*^ ions (*m/z*)	Metabolite	Molecular formula	Class	References	IPA-J	IPA-E	IPR-J	IPR-E
1.	0.66	341	nd	341: 179, 143, 125, 119, 101, 89, 71, 59.	*O*-Hexosyl-hexose (Sucrose)	C_12_H_22_O_11_	Carbohydrates	^95,^^96^	+	-	+	+
2.	1.32	331	nd	331:169bp	*O*-galloyl hexose	C_13_H_16_O_10_	Phenolic glycoside	^25^	-	-	-	+
3.	1.97	nd	166	166:120,103	phenylalanine	C_9_H_11_NO_2_	Amino acids	^97^	-	+	-	-
4.	2.78	341	nd	341:135	Caffeic acid hexoside	C_15_H_18_O_9_	Phenolic glycosides	^26^	+	-	-	-
5.	5.50	593	nd	593:407	(*epi*) Gallocatechin-(*epi*)catechin dimer	C_30_H_28_O_13_	Flavonoid	^4^	+	-	-	-
6.	6.03	583	nd	583:565,463,331, 301,259	Neomangiferin	C_25_H_28_O_16_	Xanthone	^67^	-	+	-	-
7.	6.88	593	nd	593:473,311,282	Apigenin-*C*-hexoside-*O*-hexoside	C_27_H_30_O_15_	Flavonoid	^39^	+	-	-	-
8.	6.88	593	nd	593:473,341,282	Apigenin-*C*-hexoside-*O*-hexoside isomer	C_27_H_30_O_15_	Flavonoid	^39^	+	-	-	-
9.	6.95	593	nd	593:473 BP, 503,311,282	Apigenin-*C*-hexoside-*O*-hexoside (Saponarin) (Isovitexin-7-*O*-hexoside)	C_27_H_30_O_15_	Flavonoid	^40^	+	-	-	-
10.	6.95	609	nd	609:327 bp	Luteolin-*C*-hexoside-*O*-hexoside	C_26_H_28_O_16_	Flavonoid	^49^	+	+	-	-
11.	7.09, 7.15	421	423	421:331,301,271,259, 423:351,339,303,299,273,261	Mangiferin	C_19_H_18_O_11_	Xanthone	^67,^^68^	-	+	-	-
12.	7.15	nd	611	611:395,377,329,299,287	Isoorientin-*O*-hexoside	C_27_H_30_O_16_	Flavonoid	^46,48,50^	-	+	-	-
13.	7.21	421	nd	421:331,301,271,258	Isomangiferin	C_19_H_18_O_11_	Xanthone	^29^	+	+	-	-
14.	7.52	593	nd	593:341bp	Apigenin-*C*-hexoside-*O-*hexoside isomer (Isovitexin-*O*-hexoside)	C_27_H_30_O_15_	Flavonoid	^40^	+	_	_	_
15.	7.55	647	nd	647: 459	Hydroxy-dimethoxy-flavone-acetyldeoxyhexosylhexoside	C_31_H_36_O_15_	Flavonoid	^38^	+	_	_	_
16.	7.55	609	nd	609:339,327	Luteolin-*C*-hexoside-*O*-hexoside isomer	C_26_H_28_O_16_	Flavonoid	^40^	+	+	-	-
17.	7.72	593	nd	593:399	Luteolin-*C*-hexoside-*C*-deoxyhexoside	C_27_H_30_O_15_	Flavonoid	^51^	+	-	-	-
18.	8.04	563	nd	563:443,407,383,353.	Apigenin-*C*-hexosyl-*C*- pentoside (Schaftoside)	C_26_H_28_O_14_	Flavonoid	^41^	+	+	_	_
19.	8.14	447	nd	447:371,357,327,299,285,199	Luteolin-*C*-hexoside (Orientin)	C_21_H_19_O_11_	Flavonoid	^52,^^53^	+	-	_	_
20.	8.27	593	nd	593: 299,298	Kaempferide hexosyl pentoside	C_27_H_30_O_17_	Flavonoid	^32^	+	-	-	-
21.	8.27	593	nd	593: 357,285,284,150	Kaempferol-*O*-deoxyhexosyl hexoside (Kaempferol-*O*-rutinoside)	C_27_H_30_O_15_	Flavonoid	^32,^^33^	+	-	-	-
22.	8.41	593	nd	593: 473bp	Apigenin-di-*C*-hexoside (Vicenin-2)	C_27_H_30_O_15_	Flavonoid	^42^	+	-	-	-
23.	9.29	447	nd	447:285 bp	Luteolin‐*O*‐ hexoside	C_21_H_20_O_11_	Flavonoid	^53^	+	_	_	_
24.	9.29	563	nd	563:311	Apigenin-*C*-hexoside-*O*-pentoside	C_26_H_28_O_16_	Flavonoid	^43,^^44^	+	-	-	-
25.	10.19	461	nd	461:299,283,255,240	Dihydro-methoxyisoflavone-*O*-hexoside (Tectoridin)	C_22_H_22_O_11_	Isoflavonoid	^56,^^57^	_	_	_	+
26.	10.54	301	nd	301: 139bp	Hydroxy-methoxy-phenyl-*O*-hexoside (Tachioside)	C_13_H_18_O_8_	Phenolic glycoside	^27^	-	-	-	+
27.	11.66	461	nd	461:284bp	Kaempferol-*O*-glucuronide	C_21_H_18_O_12_	Flavonoid	^35^	_	_	_	+
28.	12.24	315	nd	315: 300,272,227	Tetrahydroxy-methoxyisoflavone (Irilin D)	C_16_H_12_O_7_	Isoflavonoid	^29,^^58^	-	-	-	+
29.	12.64	299	nd	299:284bp,283,240	Trihydroxy-methoxyisoflavone (Rhamnocitrin)	C_16_H_12_O_6_	Flavonoid	^59^	-	-	-	+
30.	13.39	269	nd	269:241,225,201, 169,133	Trihydroxyisoflavone (Genistein)	C_15_H_10_O_5_	Isoflavonoid	^60^	-	-	-	+
31.	13.51	299	301	301:286,268,183,168,140. 299:284,212,200, 166.	Trihydroxy-methoxyisoflavone (Tectorigenin)	C_16_H_12_O_6_	Isoflavonoid	^61–63^	-	-	-	+
32.	13.55	301	nd	301:286,164,151, 111,87	Trihydroxy-methoxyflavanone (Hesperetin)	C_16_H_14_O_6_	Flavonoid	^54,55,98^	_	-	_	+
33.	13.82	301	nd	301:283, 240, 151,139	Dihydrokaempferide	C_16_H_14_O_6_	Flavonoid	^29,^^30^	-	-	-	+
34.	14.72	313	315	315:300,272, 313:298,283,269, 252	Dihydroxy Dimethoxy isoflavone (Irisolidone)	C_17_H_14_O_6_	Isoflavonoid	^64,^^65^	-	+	-	+
35.	14.93	313	315	315: 300, 282, 269, 254, 183, 169, 168, 133, 313: 298, 267, 255,211, 183, 167.	Dihydroxy-dimethoxy-isoflavone isomer	C_17_H_14_O_6_	Isoflavonoid	^55^	-	+	-	+
36.	16.43	329	nd	329: 229, 211, 171,139	Trihydroxy-octadecenoic acid	C_18_H_34_O_5_	Fatty acids	^53^	+	-	-	+
37.	21.18	nd	261	261: 243, 167, 121, 93	Trihydroxy-methoxy-benzophenone	C_14_H_12_O_5_	Benzophenone.	^69^	+	_	_	_
38.	24.43	nd	303	303:303,285,267,257,165,153,95	Pentahydroxy flavone (Quercetin)	C_15_H_10_O_7_	Flavonoid	^28^	_	+	+	+
39.	27.62	nd	317	317: 285,229,177,165, 153	Tetrahydroxy-methoxyflavone (Isorhamnetin)	C_16_H_12_O_7_	Flavonoid	^28^	+	-	+	+
40.	28.29	nd	433	433:271bp,227,145, 108	Apigenin-*O*-hexoside	C_21_H_20_O_10_	Flavonoid	^28,^^45^	_	_	+	_
41.	30.11	317	nd	317: 271	Trihydroxy-trimethoxy flavone (Myricetin)	C_15_H_10_O_8_	Flavonoid	^36,^^37^	-	+	-	-
42.	30.27	nd	433	433:397 ,367 , 271, 309 ,342	Apigenin-*C*-hexoside (Vitexin)	C_21_H_20_O_10_	Flavonoid	^47,^^46^	_	_	+	_
43.	31.38	nd	433	433:313,255, 121	Apigenin-*C*-hexoside isomer (Isovitexin)	C_21_H_20_O_10_	Flavonoid	^48^	_	_	+	_

**Table 2:** Tentatively identified compounds present in *I. pseudacorus* extracts.

Samples	Number of detected compounds					
	Flavonoids	Isoflavonoids	Xanthones	Phenols	Fatty acids	All
IPA-J	17	0	1	2	1	21
IPR-J	5	0	0	0	0	5
IPA-E	6	2	3	1	0	12
IPR-E	6	6	0	2	1	15

#### Phenolic acid Derivatives

Compound **(2)** displayed a pseudomolecular ion peak at *m/z* 331 [M-H]^-^. The MS^2^ profile presented a characteristic base peak at *m/z* 169 [M-H-162]^-^ corresponding to the natural loss of a hexosyl residue^25^. Compound (**2**) was tentatively identified as *O*-galloyl hexose. Compound **(4)** showed a molecular ion peak [M-H]^-^ at *m/z* 341. The MS^2^ spectrum displayed a characteristic base peak at *m/z* 135 [M-H-162-CO_2_]^-^ corresponding to decarboxylated caffeic acid after elimination of both hexose and CO_2_ molecule^26^. Consequently, compound (**4**) was recognized as caffeic acid hexoside. Compound **(26)** exhibited a molecular ion peak at *m/z* 301[M-H]^-^. Its MS^2^ profile revealed a characteristic base peak at *m/z* 139 [M-H-162]^-^ owing to the neutral loss of a hexosyl moiety^27^. Thus, compound (**26**) was putatively identified as hydroxy-methoxy-phenoxy-hexoside.

#### Flavonoids

Various flavonoids were previously detected and isolated from *I. pseudacorus* extracts either in aglycone or glycosylated forms including flavones, flavanones, flavanols, flavonols and isoflavonoids. Mass spectra can predict the skeleton of flavonoids *via* several fragmentation pathways^28^.

##### Flavanols

Compound **(5)** exhibited a molecular ion peak [M-H]^-^ at *m/z* 593 and eluted at R_*t*_ 5.50 min. The MS^2^ spectrum displayed a characteristic base peak at *m/z* 407 [M-H-168-H_2_O]^-^ was produced *via* RDA mechanism and successive loss of a water molecule^4^. This fragment is characteristic for (*epi*)gallocatechin. Thus, compound **(5)** was tentatively identified as proanthocyanidin dimer ((*epi*)gallocatechin-(*epi*)catechin dimer). Compound (**33**) revealed [M-H]^-^ with value of *m/z* 301. Its MS^2^ spectrum exhibited typical fragment ion peaks at *m/z* 283 [M-H-H_2_O]^-^ attributed to the loss of a H_2_O molecule, *m/z* 255 probably due to a subsequent loss of C_2_H_4_ moiety, and *m/z* 240 due to further loss of a methyl moiety. A fragment ion peak at *m/z* 151 was attributed to [^1,3^A-H]^-^^29,30^. Thus, compound (**33**) was tentatively identified as dihydrokaempferide and was previously isolated from the rhizomes of *I. tectorum*^31^.

##### Flavonols

Compound **(20)** demonstrated a molecular ion peak [M-H]^-^ with *m/z* 593. Its MS^2^ profile exhibited the aglycone ion [Y°]^-^ at *m/z* 299 and radical aglycone ion [Y°-H]^-^ (base peak) at *m/z* 298 produced from homolytic and heterolytic cleavage, indicating that the kaempferide aglycone. Thus, compound (**20**) was putatively recognized as kaempferide hexosyl pentoside, which is reported for the first time in the genus *Iris*^32^. Compound **(21)** showed [M-H]^-^ at *m/z* 593. Its MS^2^ spectrum revealed the aglycone ion [Y˚]^-^ at *m/z* 285 and radical aglycone ion [Y˚-H]^-^ (base peak) at *m/z* 284 corresponding to kaempferol aglycone as a result of the successive loss of hexose and deoxyhexose moieties, *m/z* 357 [M-H-146-90]^-^ due to loss of deoxyhexose and cleavage of a hexosyl unit and *m/z* 150 relative to [^1,3^A^-^-H]^-^ RDA fragmentation. Thus, compound (**21**) was identified as kaempferol-*O*-deoxyhexosyl hexoside^32,33^. Kaempferol-3-*O*-rutinoside was previously isolated from the rhizomes of *I. pseudopumila*^34^. Compound **(27)** displayed a molecular ion peak [M-H]^-^ at *m/z* 461. Its MS^2^ profile revealed distinctive radical aglycone ion for kaempferol (base peak) at *m/z* 284 [Y˚-H]^–^ due to neutral loss of one glucuronyl unit^35^. Thus, compound (**27**) was tentatively identified a kaempferol-*O*-glucuronide. This is the first report of this compound in genus ***Iris*** according to our knowledge.

Compound (**38**) showed a pseudomolecular ion peak at *m/z* 303 [M+H]^+^ and was tentatively recognized as pentahydroxyflavone (quercetin). Its MS^2^ spectrum revealed distinguished fragment ion peaks at *m/z* 285 is due to [M+H- H_2_O]^+^, *m/z* 267 corresponding to [M+H-2H_2_O]^+^, *m/z* 257 owing to [M+H-H_2_O-CO]^+^, *m/z* 165, *m/z* 153 corresponding to [^0,2^A]^+^ and [^1,3^A]^+^ , respectively, arising from C-ring cleavage. And finally a fragment ion at *m/z* 95 due to [^*0,2*^B^+^–CO-O]^+^^28^. Quercetin was previously isolated from many species *Iris* including *I. germanica*^34^. Compound (**39**) showed a molecular ion peak [M+H]^+^ at *m/z* 317. Its MS^*2*^ profile exhibited characteristic fragment ion peaks at *m/z* 285 corresponding to [M+H-CH_3_-OH]^+^, *m/z* 229 corresponding to [M+H–CH3-OH-2CO]^+^, *m/z* 177 due to [M+H–2CO–C_4_H_4_O_2_]^+^, And finally fragment ion peaks at *m/z* 165 and 153 corresponding to [^*0,2*^A]^+^, [^*1,3*^A]^+^ fragments arising from *C*-ring cleavage^28^. Thus, compound (**39**) was tentatively identified as tetrahydroxy-methoxyflavone (isorhamnetin) and previously isolated from *Iris pseudacorus*^34^.Compound (41) eluted at R_*t*_ 30.11 min exhibited a molecular ion peak [M-H]^-^ at *m/z* 317 and its distinctive fragment at *m/z* 271 due to loss of CO and H_2_O molecules^36,37^. Thus, compound (41) was tentatively identified as myricetin. It was previously isolated from *I. sanguinea*^34^.

##### Flavones

Compound (**15**) has molecular ion peak [M-H]^-^ at *m/z* 647 was tentatively identified as hydroxy-dimethoxyflavone-acetyldeoxyhexosyl-hexoside. Its MS^2^ profile showed distinctive base peak at *m/z* 459 [M-H-146-42]^-^ corresponding to loss of acetylrhamnose moiety from *C*-glycosylflavone^38^**.** 5-hydroxyl-4′,7-dimethoxyflavone-6-*C*-[*O*-(*α*-L-3′′′-acetylrhamnopyranosyl)-1→2-*β*-D-glucopyranoside was previously isolated from the leaves of *I. tectorum* Maxim^38^.

##### Apigenin derivatives

Among the identifed flavones: several **apigenin derivatives** were tentatively assigned in *I. pseudacorus extracts*. Compounds **(7, 8, 9, 14)** showed the same molecular ion peak [M-H]^-^ at *m/z* 593 but eluted at different retention times. They were all tentatively identified as isomers of apigenin-*C*-hexoside-*O*-hexoside. These compounds displayed the common fragmentation pattern of C-glycosides. Compounds (**7** and **8**) exhibited characteristic fragment ion peaks at *m/z* 473 [M-H-120]^−^ corresponding to ^*0,2*^X^-^ internal cleavage of *C*-linked hexose, *m/z* 311 [M-H-120-162]^–^ in compound (**7**) and *m/z* 341 [M-H-90-162]^–^ in compound (**8)** owing to the neutral loss of a hexosyl moiety indicating the presence of a *O*-hexosyl unit. A fragment ion peak at *m/z* 282 was due to a subsequent loss of CHO moiety. Thus, compounds (**7**) and (**8**) were tentatively identified as isomers of apigenin-*C*-hexoside-*O*-hexoside^39^**.** Isovitexin-*O*-glucoside was previously isolated from many species of the genus *Iris* including *I. setosa*^34^. Compound (**9**) showed typical fragment ion peaks at *m/z* 503 [M-H- ^0,3^X]^−^ and *m/z* 473 [(M-H)-^0,2^X]^−^. Other distinguishing fragment ion peaks at *m/z* 311 [M-H-120-162]^–^ and *m/z* 282. Thus, compound (**9**) was tentatively identified as apigenin-*C*-hexoside-*O*-hexoside (isovitexin-7-*O*-glucoside)^40^. Compound (**14**) displayed distinctive base peak at *m/z* 341 [M-H-90-162]^–^ and absence of other characteristic fragment ion peaks at *m/z* 503 [M-H-90]^−^ and *m/z* 473 [M-H-^*0,2*^X]^−^. This indicated to the presence of a terminal *O*-hexosyl unit and not directly attached to the apigenin aglycone. Thus, compound (**14**) was tentatively identified as apigenin-*C*-hexoside-*O*-hexoside isomer (Isovitexin-*X*′′-*O*-glucoside)^40^. compound (**9**) and (14) were isolated from the aerial parts of *I. ensata* and *I. sanguinea,* respectively^34^.

Compound (**18**) exhibited a molecular ion peak [M-H]^-^ at *m/z* 563. Its fragmentation pattern related to asymmetric di-*C*-glycosides. The MS^*2*^ profile showed characteristic fragment ion peaks at *m/z* 443 due to [M-H-^*0,2*^X]^-^, *m/z* 407 owing to [M-H-^*0,2*^X -2H_2_O]^**-**^, *m/z* 383 [M-H-^*0,2*^X –60]^-^ and *m/z* 353 [M-H-^*0,2*^X –90]^-^ is due to cleavage of *C*-pentosyl residue. Besides the absence of fragmentation ion peak for *C*-pentosyl unit at *m/z* 503 [M-H-60]^-^, suggested that the location of the hexose unit is at position 6. The ions at *m/z* 353 (AGly + 83) and *m/z* 383 (AGly + 113) are typical fragments of the di-*C*-glycosyl flavonoids further confirms the proposed structure. Thus, compound (**18**) was tentatively identified as apigenin-6-*C-*hexosyl-8-*C*-pentosyl (schaftoside)^41^. It was previously isolated from *I. germanica*^34^. Compound (**22**) showed a molecular ion peak [M-H] at *m/z* 593. The MS^*2*^ profile revealed the distinctive base peak at *m/z* 473 corresponding to [M-H-^*0,2*^X]. Thus, compound (**22**) was tentatively identified as apigenin di-*C*-hexoside (vicenin-2)^42^. It was previously reported in *I. ensata*^34^. Compound (**24**) displayed a molecular ion peak [M-H]^–^ at *m/z* 563. The MS^*2*^ profile revealed the distinctive base peak at *m/z* 311 [M-H-120-132]^-^ relative to ^*0,2*^X^+^ fragmentation of a *C*-hexosyl unit and loss of *O*-pentosyl unit. Presence of the base peak at *m/z* 311 (AGly + 41) indicating that the aglycone is apigenin^43,44^. Thus, compound (**24**) was tentatively identified as apigenin-*C*-hexoside-*O*-pentoside. Compound (**40**) showed a molecular ion peak [M+H]^+^ at *m/z* 433, and a base peak at *m/z* 271 [M+H-162]^+^ indicating to loss of one *O*-hexosyl unit yielding the corresponding apigenin aglycone^45^. A fragment ion at *m/z* 227 [M+H-162- CO_2_]^+^ was attributed to the loss of CO_2_ molecule and at *m/z* 145 corresponding to [^*0,4*^B–H_2_O]^+^^28^. Thus, compound (**40**) was tentatively identified as apigenin-*O*-hexoside. Apigenin-7-*O*-glucoside was previously isolated from *I. sisyrinchium* L.^34^. Compound (**42**) eluted at R_*t*_ 30.27 min showed a molecular ion peak [M+H]^+^ at *m/z* 433. The MS^*2*^ spectra exhibited characteristic fragment ion peaks at *m/z* 397 [M+H-2*H_2_O]^+^, *m/z* 367 [M+H-30-36]^+^ due to cleavage of a *C*-hexosyl unit and the loss of two H_2_O molecules, *m/z* 309 [M+H-96-28]^+^corresponding to [^*0,4*^X^+^-2H_2_O-CO], *m/z* 342 [M+H-90-H]^+^ due to ^0,3^X^+^ fragmentation in C- hexosyl unit and finally *m/z* 271 [M+H-162]^+^ giving the corresponding apigenin aglycone. Thus compound (**42**) was tentatively identified as apigenin-*C*-hexoside^46,47^. While Compound (**43**) eluted at R_*t*_ 31.38 min and exhibited the same molecular ion peak. The MS^*2*^ spectrum displayed characteristic fragment ion peaks at *m/z* 313 [M+H−120]^+^ due to ^*0,2*^X^+^ fragmentation of a *C*-hexosyl unit, *m/z* 255 [M+H−120-58]^+^ corresponding to ^*0,2*^X^+^−2CHO and *m/z* 121 due to ^*0,2*^B^+^ fragmentation arising from *C*-ring cleavage. Indicating that the B ring was monohydroxylated (apigenin derivatives). Thus, compound (**43**) was tentatively identified as apigenin-*C*-hexoside isomer^48^. Compounds **(42)** and **(43)** displayed the same molecular ion peak [M+H]^+^ at *m/z* 433 but eluted at different retention times. According to the order of elution as reported in literature^46,48^ vitexin elutes earlier than isovitexin. Thus, compound (**42**) was tentatively identified as vitexin and compound (**43**) as isovitexin. They were previously isolated from many species of genus *Iris*^34^.

##### Luteolin derivatives

Compound (**10**) showed a molecular ion peak [M-H]^-^ at *m/z* 609 and a base peak appeared at *m/z* 327 [M-H-162-120]^-^ attributed to the loss of an *O*-linked hexoside and ^*0,2*^X^+^ fragmentation of a *C*-hexosyl unit indicating a luteolin aglycone^49^. Thus, compound (**10**) was tentatively identified as luteolin-*C*-hexoside-*O*-hexoside. Isoorientin-X^′′^-*O*-glucopyranoside was previously reported in *I. sanguinea*^34^. Compound (**12**) showed a molecular ion peak [M+H]^+^ at *m/z* 611. The MS^2^ spectrum exhibited typical fragment ion peaks at *m/z* 395 [M+H−162-54]^+^ attributed to loss of *O*-hexosyl moiety and 3 H_2_O molecules, *m/z* 329 [M+H-120-162]^+^ and *m/z* 299 [M+H-150-162]^+^. This fragmentation pattern corresponded to ^*0*,2^X^+^ and ^*0,1*^X^+^ cleavage of a *C*-hexosyl unit in addition to the loss of *O*-hexosyl moiety. Another fragment ion peak at *m/z* 287 [M+H-90- CO-CO_2_]^+^ corresponded to [^*0,3*^X^+^-CO-CO_2_]**.** The base peak at *m/z* 329 (AGly + 41) indicating a luteolin aglycone. The high abundance of *m/z* 299 and absence of *m/z* 300 fragment ion indicated isoorientin (6-C-glycoside) instead of orientin (8-C-glycoside)^46,48,50^. Thus, compound (**12**) was tentatively identified as isoorientin-*O*-hexoside.

Compound (**16**) exhibited a molecular ion peak [M-H]^-^ at *m/z* 609. The MS^*2*^ profile revealed a distinctive base peak at *m/z* 339 [M-H-270]^-^ due to the loss of both 90 and 180 amu. A fragment ion peak at *m/z* 327 [M-H-162-120]^-^. The occurrence of a product ion at *m/z* 327 (AGly + 41) indicated a luteolin aglycone. Thus, compound (**16**) was tentatively identified as luteolin-*C*-hexoside-*O*-hexoside isomer^40^. Compound (**17**) exhibited a molecular ion peak [M-H]^-^ at *m/z* 593. Its MS^*2*^ profile showed the distinctive base peak at *m/z* 399 [M-H-104-90]^-^ attributed to the cleavage of a *C*- deoxyhexosyl and a *C*-hexosyl unit. This fragment ion is very characteristic for di-*C*-glycosyl flavonoids. The presence of a base peak at *m/z* 399 (AGly +113) indicated a luteolin aglycone. Thus, compound (**17**) was tentatively identified as luteolin-*C*-hexoside-*C*-deoxyhexoside^51^. It was reported for the first time in genus *Iris* to the best of our knowledge.

Compound (**19**) displayed a molecular ion peak [M-H]^-^ at *m/z* 447. The base peak at *m/z* 327 due to [M-H-^*0,2*^X]^-^ and a fragment ion at *m/z* 357 owing to [M-H-^*0,3*^X]^–^. Product ions at *m/z* 299 [M-H-^*0,2*^X^-^-CO]^-^ was detected to the subsequent loss of a carbonyl group, and *m/z* 285 corresponding to [M-H-^*0,3*^X^-^-CO-CO_2_]^- 52^. The differentiation between isoorientin and orientin was made considering the MS^*2*^ fragment ions at *m/z* 429 [M–H–H_2_O]^−^ and *m/z* 411 [M–H–2*H_2_O]^−^, which are characteristic for isoorientin and are absent in orientin. In addition, the base peaks at *m/z* 357 and *m/z* 327 were characteristic for isoorientin and orientin, respectively^53^. Thus, compound (**19**) was identified as luteolin-8-*C*-hexoside (orientin). It was previously reported in many species of genus *Iris*^34^.Compound (**23**) exhibited a molecular ion peak [M-H]^-^ at *m/z* 447 and strong fragment ion at *m/z* 285 [M-H-162]^–^ corresponding to the loss of a hexosyl moiety. Thus, compound (**23**) was annotated as luteolin-*O*-hexoside^53^. Luteolin-7-*O*-glucoside was previously isolated from the aerial parts of *I. sisyrinchium*^34^.

##### Flavanones

Compound (**32**) presented a molecular ion peak [M-H]^-^ at *m/z* 301. Its MS^2^ profile exhibited characteristic fragment ion peaks at *m/z* 286 [M-H-CH_3_]^-^ due to loss of a methyl moiety, *m/z* 164 corresponds to [M-H-C_7_H_5_O_3_]^-^ and *m/z* 151 produced by RDA cleavage fragmentation at 2, 3-position of C-ring in the Flavanone^54^. Besides typical fragments were observed at *m/z* 111, *m/z* 87^55^. Thus, compound (**32**) was identified as hesperetin. It was previously reported in the rhizomes of *I. tectorum* Maxim^34^.

##### Isoflavonoids.

They comprise a huge, distinguished class of secondary metabolites isolated from the genus *Iris*^34^. Compound (**25**) showed a deprotonated molecular ion [M-H]^-^ at *m/z* 461. The MS^*2*^ profile exhibited characteristic fragment ion peaks at *m/z* 299 [M-H-162]^-^ corresponding to the loss of *O*-hexosyl moiety, *m/z* 284 [M-H-162-CH_3_]^-^ and a radical product ion at *m/z* 283 [M-H-162-CH_3_-H]^-·^ with high relative abundance. Other distinctive fragment ion peaks at *m/z* 255 corresponded to a subsequent loss of CO molecule from *m/z* 283 while *m/z* 240 attributed to the loss of CO_2_ molecule from *m/z* 284^56,57^. Thus, compound (**25**) was tentatively identified as tectoridin. It was previously reported in the rhizomes *I. spuria*^34^.

Compound (**28**) displayed a molecular ion peak [M-H]^-^ at *m/z* 315. Its MS^2^ profile showed the diagnostic base peak at *m/z* 300 owing to [M-H-CH_3_]^-^ and two characteristic fragments at *m/z* 272 [M-H-CH_3_-CO]^-^and *m/z* 227 [M-H- CH_3_-CO-CO_2_]^-^ due to a subsequent loss of CO, followed by CO_2_^29,58^. Thus, compound (**28**) was tentatively identified as tetrahydroxy-methoxyisoflavone (irilin D). Compound **(29)** exhibited a pseudomolecular ion peak at *m/z* 299 [M-H]^–^. Its MS^*2*^ profile exhibited characteristic fragment ion peaks at *m/z* 284 (base peak) corresponding to [M-H-CH_3_]^–^_,_ the radical aglycone ion at *m/z* 283 [Y˚-H]^–^ and *m/z* 240 [M-H-CH_3_-CO_2_]^-^ due to a subsequent loss of carbon dioxide. Thus, compound **(29)** was tentatively identified as trihydroxy-methoxyisoflavone (rhamnocitrin)^59^. Compound (30) showed a deprotonated molecular ion [M-H]^-^ at *m/z* 269. Its MS^*2*^ profile exhibited characteristic fragment ion peaks at *m/z* 241 due to [M-H-CO]^-^, *m/z* 225 corresponding to [M-H-CO_2_] ^–^ followed by a subsequent loss of 2 CO molecules to yield *m/z* 169 as a fragment ion. Besides, fragment ions at *m/z* 201 attributed to [M-H-C_3_O_2_]^-^ and *m/z* 133 due to [^*0,3*^B^-^] *C-*ring cleavage^60^. Thus, compound (**30**) was tentatively identified as genistein. Noteworthy, compound (**28**), (**29**), (**30**) which was previously reported in *I. tectorum*^34^.

Compound (**31**) displayed a molecular ion peak at *m/z* 301 in positive ionization mode and *m/z* 299 in negative ionization mode. The MS^*2*^ profile showed a characteristic fragment ion peak at *m/z* 286 due to [M+H-CH_3_]^+^ followed by a subsequent loss of H_2_O molecule to yield *m/z* 268 as a product ion. A fragment ion at *m/z* 183 was detected corresponding to [^*1,3*^A]^+^ RDA fragmentation arising from *C*-ring cleavage followed by a subsequent loss of CH_3_ to give the base peak at *m/z* 168. Aside, a product ion was detected at *m/z* 140 corresponding to [^*1,4*^A]^+^^61,62^. In the same context, inspecting fragmentation in the negative ion mode, the MS^*2*^ spectrum showed a diagnostic base peak at *m/z* 284 due to [M-H-CH_3_]^-^, *m/z* 212 corresponding to [284-CO_2_-CO]^-^ and *m/z* 166 attributed to [^*0,3*^A]^-^ arising from *C*-ring cleavage^63^. Thus, compound (**31**) was tentatively identified as trihydroxy-methoxyisoflavone (tectorigenin). Compound **(34)** showed a molecular ion peak at *m/z* 315 in positive ionization mode and *m/z* 313 in negative ionization mode. The MS^*2*^ spectrum showed a diagnostic base peak at *m/z* 300 is due to [M+H-CH_3_]^+^. A characteristic fragment ion peak at *m/z* 272 corresponding to [M+H-CO]^+^. The MS^2^ profile in negative ionization mode displayed a diagnostic base peak at *m/z* 298 owing to [M-H-CH_3_]^-^ followed by a subsequent loss of another CH_3_ moiety to yield *m/z* 283. Besides characteristic fragment ion peaks at *m/z* 269 due to [M-H-CH_3_-CHO]^-^, *m/z* 252 attributed to [M-H-C_2_H_5_O_2_]^-^^64,65^. Thus, compound (**34**) was identified as dihydroxy-dimethoxyisoflavone (irisolidone). It was previously reported in the rhizomes *I. germanica*^34^.Compound (**35**) displayed a molecular ion peak at *m/z* 315 in the positive ionization mode and *m/z* 313 in the negative ionization mode. The MS^*2*^ profile in the positive ionization mode showed a characteristic fragment ion peak at *m/z* 300 due to [M+H-CH_3_]^+^ followed by a subsequent loss of H_2_O molecule to yield *m/z* 282 as a product ion. Other fragment ion peaks were detected at *m/z* 269 corresponding to [M+H-2*CH_3_-O]^+^, *m/z* 254 attributed to [M+H-CH_3_O_2_]^+^, *m/z*183 due to [M+H-C_5_H_6_O_4_]^+^ and *m/z* 168 corresponding to [^*1,3*^A]^+^ RDA fragmentation arising from *C*-ring cleavage. The MS^2^ spectrum in the negative ionization mode showed a diagnostic base peak at *m/z* 298 due to [M-H-CH_3_]^-^ and a fragment ion at *m/z* 267 corresponding to [M-H-2CH_3_-O]^-^, *m/z* 255 attributed to [M-H-C_3_H_6_O] ^–^ and *m/z* 211 due to [M-H-C_4_H_6_O_3_]^-^^55^. Thus, compound (**35**) was identified as dihydroxy-dimethoxyisoflavone isomer (5,7-Dihydroxy-2',6-dimethoxyisoflavone). Compound (**31**) and (**35**) was previously isolated from *I. pseudacorus* rhizomes^34^.

#### Xanthone Derivatives

*Iris* species have been known as a rich source of xanthones^66^. Mangiferin, is the most abundant natural glycosylated xanthone and widely reported in literature. Compound (**6**) showed a molecular ion peak [M-H]^–^ at *m/z* 583. Its MS^2^ profile exhibited characteristic fragment ion peaks at *m/z* 565 corresponding to [M-H-H_2_O]^−^ , *m/z* 463 corresponding to [M-H-^*0,2*^X]^−^ and *m/z* 331 [M-H-162-90]^−^ due to ^*0,3*^X^-^ fragmentation in *C*-linked hexose and loss of *O*-hexosyl moiety. The base peak at *m/z* 301 was assigned to [M_−_H_−_162_−_^*0,2*^X^-^]^−^ and *m/z* 259 [M-H-(2 × 162)]^-^ owing to the loss of two hexosyl units^67^. Thus, compound (**6**) was tentatively identified as neomangiferin. It was previously reported in *I. dichotoma*^34^. Compound (**11**) showed a molecular ion peak [M+H]^+^ at *m/z* 423. Its MS^*2*^ profile exhibited typical fragment ion peaks at *m/z* 351 attributed to [M+H-4H_2_O]^+^, *m/z* 339 [M+H-30-54]^+^ due to [^2,3^X^+^ -3*H2O], *m/z* 303, *m/z* 273 corresponding to [M+H-^*0,2*^X]^+^ , [M+H-^*0,1*^X]^+^, respectively and *m/z* 261 [M+H-162]^+^ due to loss of a hexosyl moiety^68^. Thus, compound (**11**) was tentatively identified as mangiferin. Besides in the positive ionization mode, the fragmentation pattern of mangiferin (**11**) showed a stronger relationship than with isomangiferin (**13**), because the base peak ion in mangiferin was noticed at *m/z* 273 (due to the location of *C*-2 glucose on the di benzo-*γ*-pyrone skeleton) but in isomangiferin the base peak ion was observed at *m/z* 303. In the negative ionization mode, compound (**11**) exhibited a deprotonated molecular ion [M-H]^–^ at *m/z* 421. Its MS^2^ profile displayed characteristic fragment ion peaks at *m/z* 331, *m/z* 301, *m/z* 271 corresponding to ^*0,3*^X^-^,^*0,2*^X^-^, ^*0,1*^X^-^ fragmentation in *C*-linked hexose respectively, and *m/z* 259 due to [M-H-162]^-^^67^. Mangiferin and isomangiferin were previously isolated from *I. pseudacorus* leaves^34^*.*

#### Fatty Acid Derivatives

Compound **(36)** eluted at R_*t*_ 16.43 min displayed a molecular ion peak [M-H]^–^ at *m/z* 329. Its MS^*2*^ profile revealed characteristic fragment ion peaks at *m/z* 229 corresponding to the loss of the end-group HOCHCH(CH_2_)_3_CH_3_ from an oxylipin molecule. A product ion at *m/z* 211 was attributed to [M-H-C_6_H_12_O_2_]^*-*^ and *m/z* 171 relative to [M-H-C_9_H_14_-H_2_O]^-^^53^. Thus, compound (**36**) was identified as trihydroxy-octadecenoic acid.

#### Identification of Other Compounds

Compound **(37)** eluted at R_*t*_ 21.18 min displayed a molecular ion peak [M+H]^+^ at *m/z* 261. Its MS^*2*^ spectrum displayed characteristic fragment ion peaks at *m/z* 243 due to [M+H-H_2_O]^+^, *m/z* 167 and *m/z* 93 corresponding to ^*1,3*^A^+^ and ^*1,3*^B^+^, respectively. A product ion at *m/z* 121 relative to [^*1,3*^A^+^- H_2_O-CO]^+^^69^. Thus, compound (**37**) was putatively identified as trihydroxy-methoxybenzophenone which was previously reported in the rhizomes of *I. adriatica*^70^ and *I. pallida*^71^.

### In Vitro biological evaluation

#### In Vitro evaluation of antioxidant activity using DPPH assay

The antioxidant potential of *I. pseudacorus* extracts was evaluated using DPPH assay (Table 3). *I. pseudacorus* rhizomes extracts (IPR-J and IPR-E) exhibited the highest radical scavenging activity with % inhibition 75.84, 60.75 at a concentration of 125 μg/mL and IC_50_ values of 40.89 µg/mL, 97.97 µg/mL, respectively. Trolox was used as positive control exhibiting IC_50_ value at 14.59 µg/mL. While the lowest values were shown by aerial part methanol extracts of IPA-J and IPA-E with % inhibition 30.43 and 33.93 at concentration 125 μg/mL respectively.

**Table 3:** DPPH, *α*-glucosidase, lipase and tyrosinase inhibitory activity of *I. pseudacorus* extracts.

Sample	DPPH		*α*-glucosidase		Lipase		Tyrosinase	
	%Inhibition	IC_50_ µg/mL	%Inhibition	IC_50_ µg/mL	%Inhibition	IC_50_ µg/mL	%Inhibition	IC_50_ µg/mL
IPA-J	30.43	NC	NA	NC	92.91	2.22±0.13	NA	NC
IPR-J	75.84	40.89± 0.92	99.96	18.52± 0.75	97.67	2.35± 0.03	9.43	NC
IPA-E	33.93	NC	NA	NC	95.99	0.42± 0.01	NA	NC
IPR-E	60.75	97.97± 3.44	67.70	57.89± 2.32	93.63	4.81± 0.09	10.34	NC

The results are calculated as mean *±* SD, n=3.

NA, not active; NC, not calculated; IPA-J, Japanese *I. pseudacorus* aerial parts; IPR-J, Japanese *I. pseudacorus* rhizomes; IPA-E, Egyptian *I. pseudacorus* aerial parts; IPR-E, Egyptian *I. pseudacorus* rhizomes.

#### Enzyme inhibitory activity

In the current study, the enzyme inhibitory activity of *I. pseudacorus* extracts was assessed in vitro against tyrosinase, *α*-glucosidase and lipase enzymes. Results are displayed in Table 3.

##### In Vitro anti-hyperglycemic evaluation using *α*-glucosidase enzyme assay

The anti-hyperglycemic activity of *I. pseudacorus* extracts (IPA-J, IPR-J, IPA-E, and IPR-E) was evaluated in vitro using *α*-glucosidase inhibitory assay. Results revealed that *I. pseudacorus* rhizomes methanol extracts (IPR-J and IPR-E) exhibited the highest *α*-glucosidase inhibitory activity displaying percentage inhibition of 99.96, 67.70 at a concentration of 100 μg/mL and IC_50_ values are 18.52 µg/mL, 57.89 µg/mL, respectively being more potent as compared to acarbose with IC_50_ value of 362.088 µg/mL (Table 3). On the other hand, the aerial part methanol extracts of *I. pseudacorus* (IPA-J and IPA-E) showed no *α*-glucosidase inhibitory activity.

##### In Vitro anti- hyperlipidemic activity using pancreatic lipase enzyme assay

The antihyperlipidaemic activity of *I. pseudacorus* extracts (IPA-J, IPR-J, IPA-E, IPR-E) was evaluated in vitro for potential pancreatic lipase inhibitory activity. As shown in Table 3, all extracts of *I. pseudacorus* exerted significant lipase inhibitory activity and were potent compared to cetilistat used as positive control. The % inhibition of IPR-J, IPR-E, IPA-J, IPA-E was 97.67, 93.63, 92.91 and 95.99, respectively at a concentration of 25 μg/ml. Besides, IC_50_ values were 2.35 µg/mL, 4.81 µg/mL, 2.22 µg/mL and 0.42 µg/mL respectively compared to cetilistat with IC_50_ value of 7.47 µg/mL.

##### In Vitro anti-melanogenesis activity using tyrosinase enzyme assay

The tyrosinase inhibitory activity of the *I. pseudacorus* extracts (IPA-J, IPR-J, IPA-E and IPR-E) was evaluated in vitro. As shown in Table 3, no anti-melanogenesis activity was observed for all *I. pseudacorus* extracts up to 500 µg/mL. Arbutin was used as a positive control and displayed an IC_50_ value of 120 µg/mL.

### Molecular docking studies

#### Molecular docking

Molecular docking studies were conducted for the major identified compounds in *I. pseudacorus* extracts within the active sites of human *α*-glucosidase (HAG) and human pancreatic lipase (HPL) (Table 4).

**Table 4:** Free binding energies (∆G) of the major identified compounds in *I. pseudacorus* extracts within the active sites of human *α*-glucosidase (HAG) and human pancreatic lipase (HPL) using molecular docking and expressed in kcal/mol.

Compound	C-docker energy ∆G (Kcal/mol)	
	*α*-Glucosidase	Pancreatic lipase
Quercetin (**38**)	− 44.02	− 37.35
6-*O*-Galloylglucose (**2**)	− 43.09	− 33.49
Irilin D (**28**)	− 34.92	− 31.51
Rhamnocitrin (**29**)	− 30.66	− 30.49
Kaempferol-*O*-glucuronide (**27**)	− 30.11	− 18.71
Tectorigenin (**31**)	− 28.22	− 25.65
Genistein (**30**)	− 27.86	− 20.63
Mangiferin (**11**)	− 27.81	− 22.55
Luteolin-7-*O*‐ glucoside (**23**)	− 27.74	− 19.90
Apigenin-7-*O*-glucoside (**40**)	− 26.13	− 13.62
5,7-Dihydroxy-2,6-Dimethoxyisoflavone (**35**)	− 25.18	− 20.47
Dihydrokaempferide (**33**)	− 24.94	− 23.09
Irisolidone (**34**)	− 24.85	− 22.58
Orientin (**19**)	− 24.49	− 22.72
Isovitexin (**43**)	− 23.11	− 22.19
Isomangiferin (**13**)	− 20.69	− 15.67
Vitexin (**42**)	− 19.54	− 18.71
tectoridin (**25**)	− 17.53	− 10.40
Isoorientin 6''-glucoside (**10**)	f.d.	− 6.96
Schaftoside (**18**)	f.d.	− 5.82
Vicenin-2 (**22**)	f.d.	− 7.01
Neomangiferin (**6**)	f.d.	6.34
Isovitexin-7-*O*-glycoside (**9**)	f.d.	9.42
Acarbose (3TOP co-crystallized inhibitor)	− 59.61	–
Cetilistat	–	− 51.24
Methoxyundecylphosphinic acid (1LPB co-crystallized inhibitor)	–	− 30.34

*f.d.* failed to dock.

Positive values indicate unfavorable interaction.

**Figure 5:**
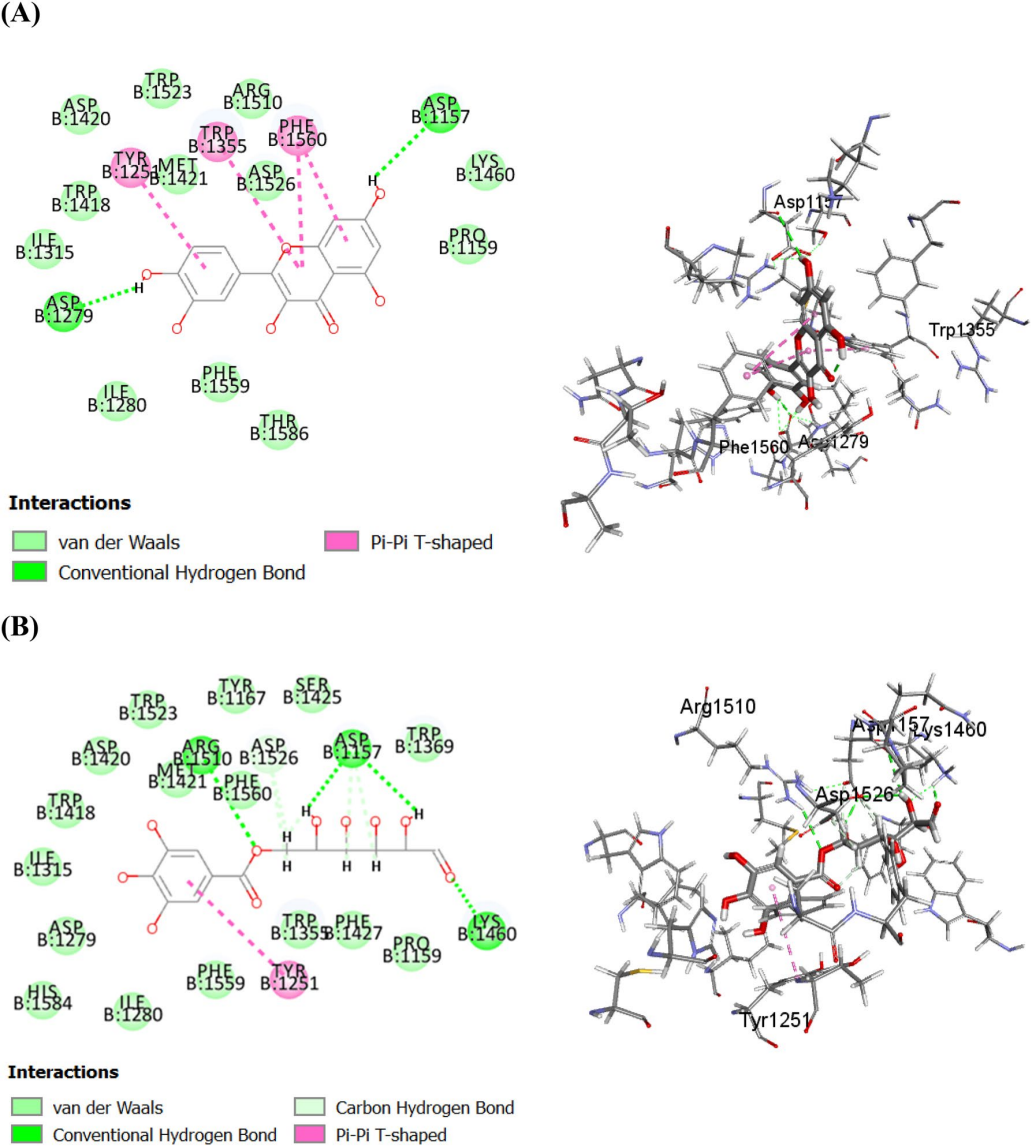

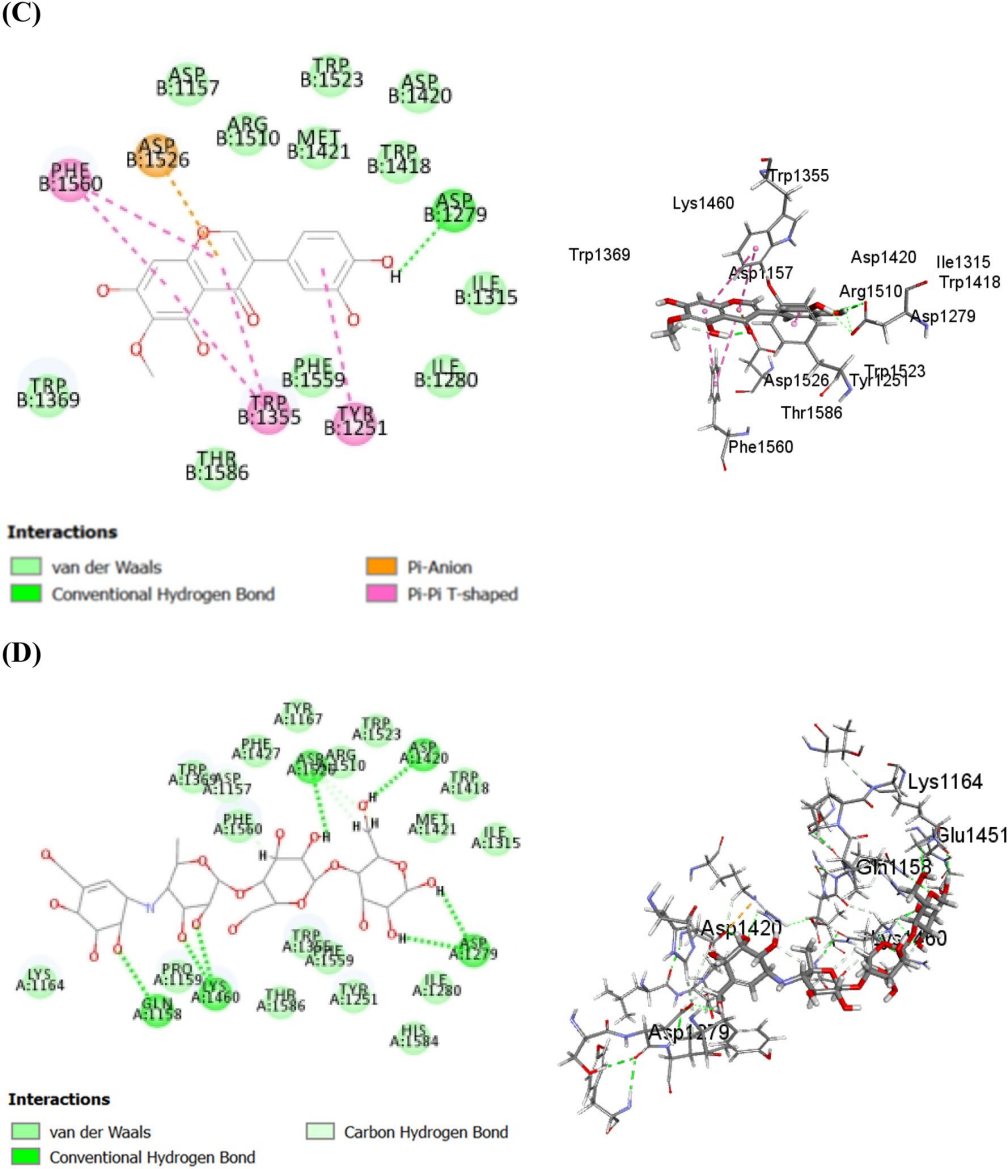
2D and 3D binding modes of quercetin (**A**), galloyl glucose (**B**), Irilin D (**C**), and acarbose (**D**) within the active sites of human α-glucosidase (HAG).

Regarding human ***α***-glucosidase, quercetin was the top hit compound, followed by galloyl glucose and then irilin D displaying free binding energy equals to − 44.02, − 43.09 and − 34.92 kcal.mol^−1^, respectively, approaching that of acarbose (the co-crystallized inhibitor) with ∆G=− 59.61 kcal.mol^−1^. Quercetin showed firm binding to HAG with formation of π − π hydrophobic interaction with Phe1560, Tyr1251 and Trp1355 as well as two conventional hydrogen bonds with Asp1157 and Asp1279 (Fig. 5A). Besides, galloyl glucose formed one π − π bond with Tyr1251, four conventional hydrogen bonds with Asp 1157, Lys1460 and Arg1510 and three C–H bonds with Asp1157 and Asp1526 (Fig. 5B). In the same context, irilin D was firmly bound to the catalytic residues of HAG by four π − π bonds with Phe1560, Tyr1251 and Trp1355; one conventional hydrogen bond with Asp1279 and one anion-π interaction with Asp1526 (Figure 5C). Noteworthy, acarbose displayed favorable interaction within the active sites of human ***α***-glucosidase forming seven conventional hydrogen bonds with Asp 1526, Asp 1420, Asp1279, Lys1460 and Gln1158 and two C–H bonds with Asp1526 (Figure 5D).

Similarly, molecular docking of the major metabolites identified in *I. pseudacorus* extracts within the active sites of human pancreatic lipase revealed favorable binding where quercetin, galloyl glucose and irilin D exhibited the best fitting scores with ∆G equals to − 37.35, − 33.49, − 31.51 kcal.mol^−1^ approaching that of cetilistat (an irreversible pancreatic lipase inhibitor) with ∆G = − 51.24 kcal.mol^−1^ and methoxyundecylphosphinic acid (MUP), the co-crystallized inhibitor and a reversible pancreatic lipase inhibitor, with ∆G = − 30.34 kcal.mol^−1^.

**Figure 6:**
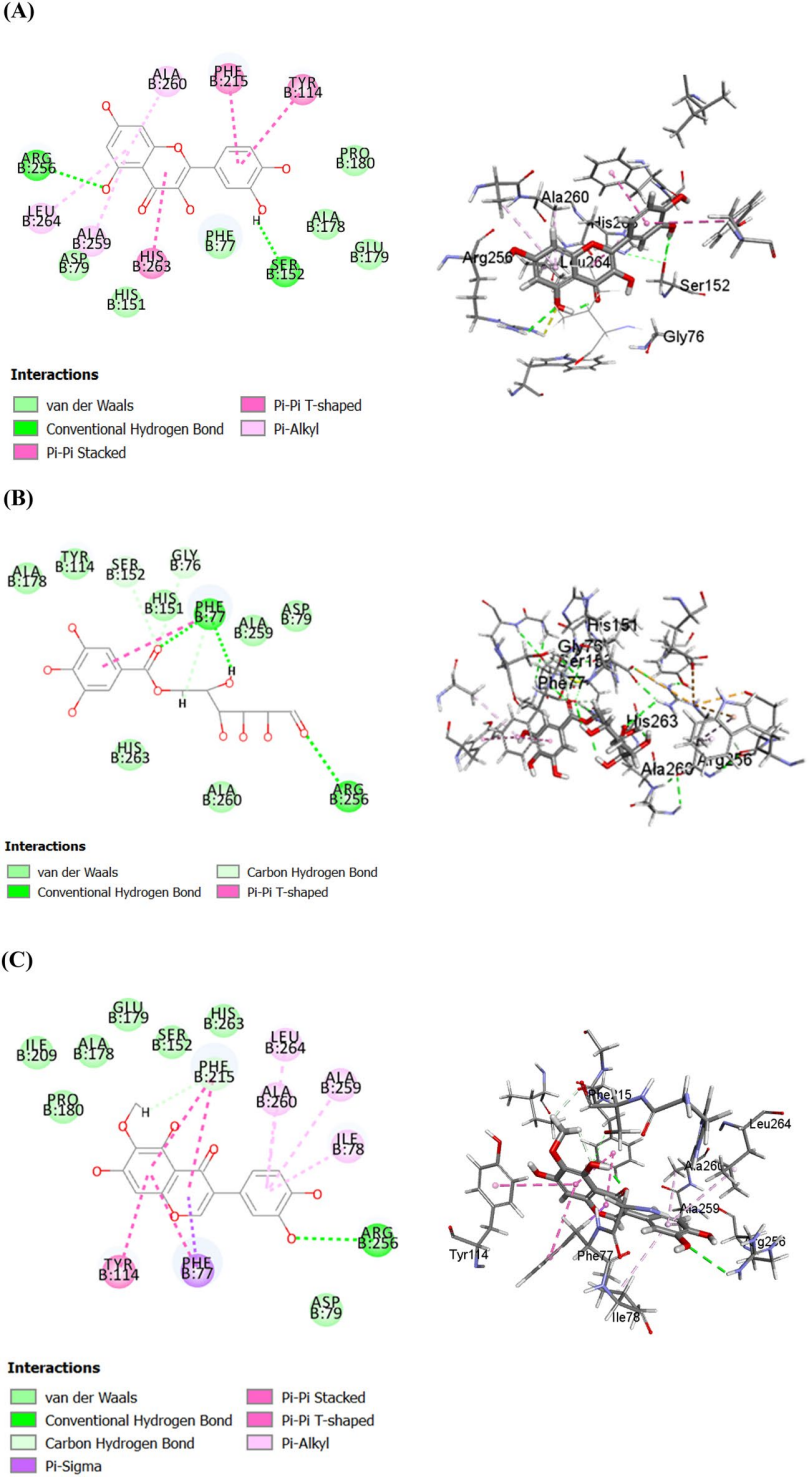

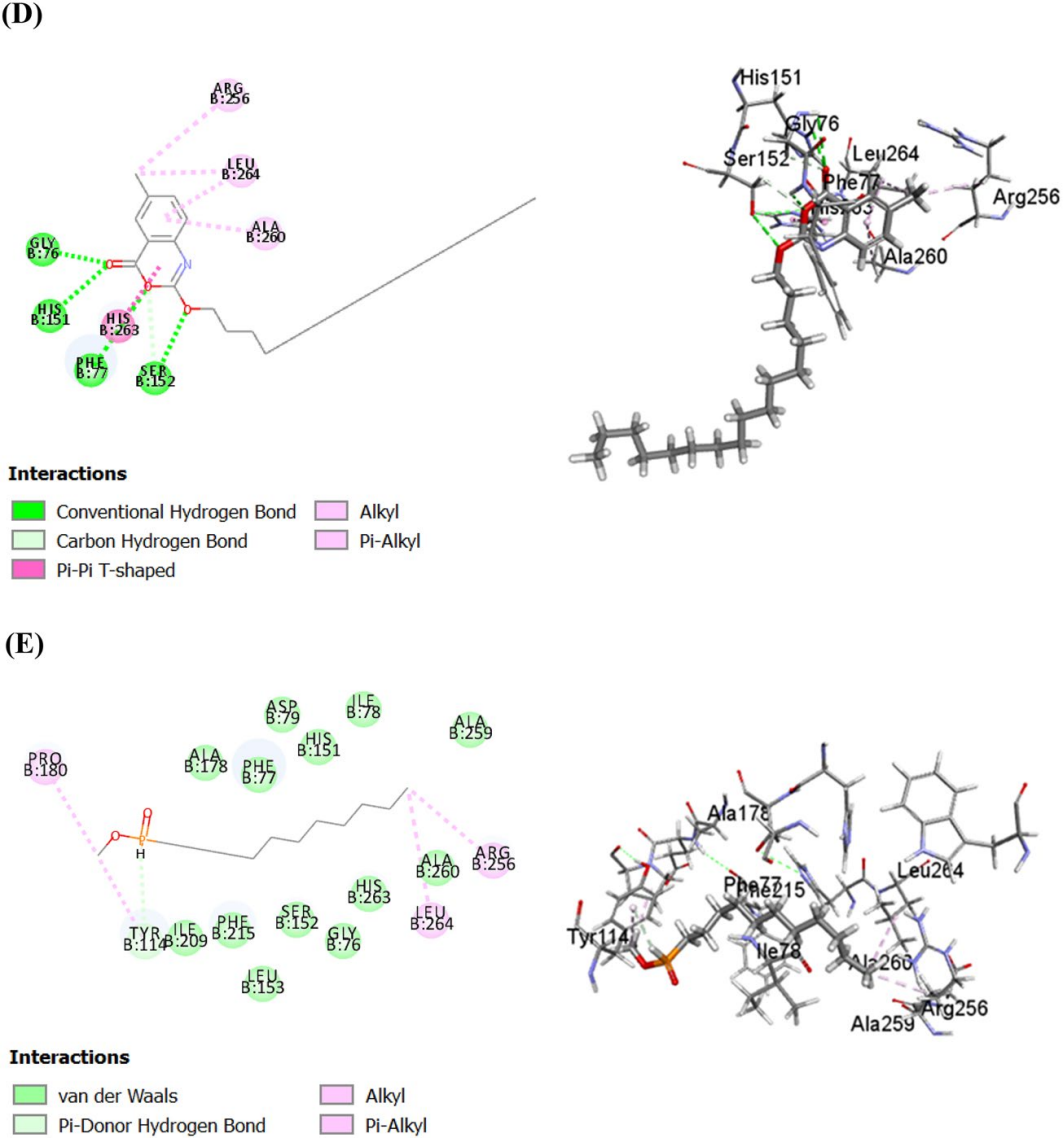
2D and 3D binding modes of quercetin (**A**), galloyl glucose (**B**), Irilin D (**C**), cetilistat (**D**), and methoxyundecylphosphinic acid MUP (1LPB co-crystallized inhibitor) (**E**) within the active sites of human pancreatic lipase (PL).

Quercetin showed strong π − π hydrophobic interaction with Phe215, Tyr114 and His263 in addition to three π-alkyl bonds with Leu264, Ala259 and Ala260 and two conventional hydrogen bonds with Arg256 and Ser152 (Fig. 6A). Meanwhile, galloyl glucose forms three conventional hydrogen bonds with Arg256 and Phe77; one π − π bond with Phe77 and three C–H bonds with Ser152, Gly76 and Phe77 (Fig. 6B). Besides, irilin D formed one conventional hydrogen bond with Arg256; four π-alkyl bond with Leu264, Ala259, Ala260, Ile78; one C–H bond with Phe215; one π-δ bond with Phe77 and four π − π bond with Tyr114, Phe 215 and Phe 77 (Fig. 6C). In the same context, cetilistat showed firm affinity to HPL with formation of five intermolecular hydrogen bonds with Gly76, Asp79, Ser152, Phe77 and His151 and one π − π bond with His263 (Fig. 6D). Furthermore, MUP formed two alkyl bonds with Arg 25 and Leu264; one π -alkyl bond with Pro180 and one π- donor hydrogen bond with Tyr114 (Fig. 6E). Noteworthy, Van der Waals forces represented a common interaction between all of these compounds and the amino acid residues present in the binding site of HPL and HAG enzymes.

#### ADMET prediction

**Figure 7:**
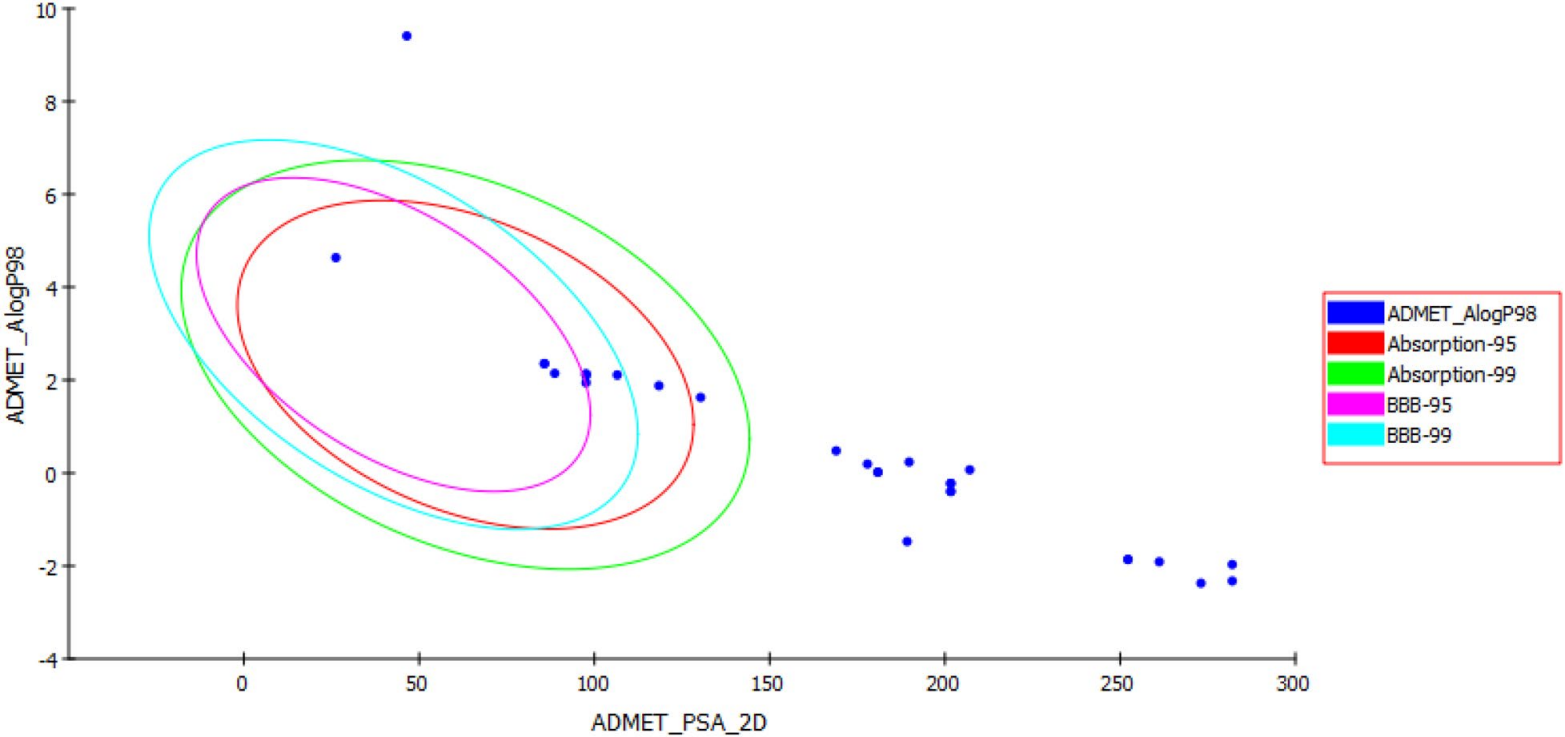
ADMET plot for bioactive metabolites identified in *I. pseudacorus* extracts displaying 95% and 99% confidence limit ellipses corresponding to blood-brain barrier (BBB) and human intestinal absorption models.

The purpose of ADMET prediction is to examine whether *I. pseudacorus* phytoconstituents possess drug-like properties or not. It is an essential step in the pharmaceutical R&D development. Descriptors of ADMET plot revealed that several compounds identified in *I. pseudacorus* extracts showed adequate intestinal absorption and aqueous solubility, thus, inferring good oral absorption. Moreover, they displayed low and undefined penetration through blood-brain barrier (BBB) and hence very low possibility for central nervous system (CNS) toxicity^72^. Besides, they were non-inhibitors for CYP2D6, thus, they could be easily excreted in phase 1 metabolism. Unfortunately, most of these constituents exhibited plasma protein binding PPB and hence fewer chances to reach to their targets (low bioavailability). Noteworthy, compounds (**28**, **29**, **30**, **31**, **33**, **34**, **35** and **38**) showed excellent intestinal absorption, as evidenced by their allocation in the 99% absorption ellipse (Fig. 7). Moreover, all compounds including cetilistat and acarbose exhibited very good solubility except compounds (**6**, **9**, **10**, **18** and **22**). Besides, compounds (**29**, **30**, **31**, **33**, **34** and **35**) showed low penetration through BBB and hence were positioned inside the 99% BBB eclipse, concomitantly other compounds had undefined level of penetration and hence were positioned outside the 99% BBB eclipse (Fig. 7). Additionally, all compounds except (**2**, **18** and **22**) displayed more than 90% PPB. However, compounds (**2**, **6**, **9**, **10**, **11**, **18**, **22**, **27**, **34** and **35**) including MUP were non-inhibitors for CYP2D6. Nevertheless, all compounds exhibited certain hepatotoxicity (Table 5)^73^.

**Table 5:** Absorption, distribution, metabolism, excretion, and toxicity (ADMET) properties of major metabolites identified in *Iris pseudacorus* extracts.

Compound name	BBB level	Absorption level	Solubility level	Hepato-toxicity	CYP2D6	PPB level	AlogP98	PSA 2D
6-*O*-Galloylglucose (**2**)	4	3	4	1	0	0	0	189.24
Apigenin-7-*O*-glucoside (**40**)	4	3	3	1	1	2	0	168.984
Luteolin-7-*O*‐ glucoside (**23**)	4	3	3	1	1	2	0	189.799
Dihydrokaempferide(**33**)	3	0	3	1	1	2	0	97.607
Genistein (**30**)	3	0	3	1	1	2	0	88.677
Irilin D (**28**)	4	0	3	1	1	2	0	118.422
Irisolidone (**34**)	3	0	3	1	0	2	0	85.722
Isomangiferin (**13**)	4	3	3	1	1	2	0	201.684
Isoorientin 6''-Glucoside (**10**)	4	3	2	1	0	2	0	281.991
Isovitexin (**43**)	4	3	3	1	1	2	0	180.869
Isovitexin-7-O-glycoside (**9**)	4	3	2	1	0	2	0	261.176
Kaempferol 7-O-Glucuronide (**27**)	4	3	3	1	0	2	0	207.1
Mangiferin (**11**)	4	3	3	1	0	2	0	201.684
Neomangiferin (**6**)	4	3	1	1	0	2	0	281.991
Orientin (**19)**	4	3	3	1	1	2	0	201.684
Quercetin (**38**)	4	1	3	1	1	2	0	130.308
Rhamnocitrin (**29)**	3	0	3	1	1	2	0	97.607
Schaftoside (**18**)	4	3	2	1	0	0	0	252.246
Tectoridin (**25**)	4	3	3	1	1	2	0	177.914
Tectorigenin (**31**)	3	0	3	1	1	2	0	97.607
Vicenin-2 (**22**)	4	3	2	1	0	0	0	273.061
Vitexin (**42**)	4	3	3	1	1	2	0	180.869
5,7-Dihydroxy-2',6-Dimethoxyisoflavone (**35**)	3	0	3	1	0	2	0	85.722
Cetilistat	4	3	1	0	1	2	0	46.484
Mup	0	0	3	0	0	1	1	26.23
Acarbose	4	3	1	1	0	0	0	328.062

0, 1, 2, 3, and 4 denote very high, high, medium, low, and undefined penetration *via* BBB respectively. 0, 1, 2, and 3 signify good, moderate, poor, and very poor intestinal absorption, respectively. Aqueous solubility: 0, 1, 2, 3, 4, and 5 show extremely low, very low but possible, low, good, optimal, and too soluble, respectively. Hepatotoxicity: 0, non-toxic; 1, toxic. CYP2D6, cytochrome P450-14DM inhibition: 0, non-inhibitor; 1, inhibitor. PBB, plasma protein binding: 0, less than 90%; 1, more than 90%. AlogP98, atom-type partition coefficient (ALogP98). PSA 2D, 2D polar surface area in A^2^.

## Discussion

Metabolic profiling of Egyptian and Japanese *I. pseudacorus* aerial parts and rhizomes crude extracts was carried out using UPLC-ESI-MS/MS analysis to obtain better insight into the phytochemical profile that may contribute to the studied biological activities. Forty-three compounds were tentatively identified herein. The identified metabolites belonged to various chemical classes including 33 flavonoids, 3 xanthones, 3 phenolic acid derivatives, an amino acid, a sugar, a benzophenone, and a fatty acid. Xanthones were detected only in the aerial parts of each cultivar.

The normal metabolic processes produce free radicals that possess unpaired electrons which are extremely reactive and result in cell injury^74^. In the current study the antioxidant potential of *I. pseudacorus* extracts was evaluated using DPPH assay. The mechanism of DPPH radical scavenging assay relies on electron transfer reaction, wherein DPPH acts as a radical^3^. The ability of *I. pseudacorus* extracts to scavenge free radicals could be correlated to the high polyphenolic content of this plant (mainly isoflavonoids and flavonoids), which are accumulated mainly in the rhizomes of the two cultivars and easily donate proton ions from their phenolic hydroxyl groups to scavenge free radicals into less reactive radicals. The tentatively identified constituents in IPR-J and IPR-E were previously reported to exert significant antioxidant activity^75,76^. The promising antioxidant activity of IPR-E could be attributed to tectoridin, tectorigenin, quercetin, irisolidone, genistein, rhamnocitrin, irilinD, dihydrokaempferide and 5,7-dihydroxy-2',6-dimethoxyisoflavone identified in the rhizome extract. In the same context vitexin, isovitexin and quercetin could be responsible for the observed activity of IPR-J^75,76^. The free radical scavenging abilities of tectorigenin (a metabolite formed by tectoridin metabolism *via* intestinal microflora) and found in IPR-E, were previously investigated in vivo. Tectorigenin scavenged intracellular free radicals and reduced lipid peroxidation in Chinese hamster lung protecting the viability of fibroblast (V79-4) cells exposed to hydrogen peroxide *via* the stimulation of extracellular signal regulated kinase (ERK)^77^. Besides, irisolidone exhibited promising antioxidant activity with IC_50_ value 12.62 μg/mL compared to propyl gallate (IC_50_ 6.72 μg/mL)^78^. In addition, vitexin displayed promising free radical scavenging activity with IC_50_ value 31.4 μg/mL compared to trolox IC_50_ 17.3 μg/mL^79^. Genistein was considered an effective antioxidant agent with IC_50_ value at 1.89 ± 0.16 μg/mL for DPPH compared to trolox IC_50_ value at 0.0247 ± 0.005 μg/mL^80^.

Several enzymes including *α*-glucosidase, lipase, and tyrosinase are considered as potential targets for lessening symptoms of diabetes mellitus, obesity and skin disorders, respectively^1^. Treatment of diabetes and its complications could be achieved by inhibiting key digestive enzymes as α-glucosidase that participate in starch digestion^6^. The anti-hyperglycemic activity of *I. pseudacorus* extracts (IPA-J, IPR-J, IPA-E, and IPR-E) was evaluated in vitro using *α*-glucosidase inhibitory assay. The promising anti-hyperglycemic activity of IPR-J and IPR-E could be attributed to richness in flavonoids, isoflavonoids and phenolic acids. These secondary metabolites were previously shown to possess anti-hyperglycemic activity *via* acting as potent *α*-glucosidase inhibitors^75^. Noteworthy, studies for *α*-glucosidase inhibitory activity of various *Iris* species are still scarce. The potent *α*-glucosidase inhibitory activity of IPR-E could be attributed to tachioside^81^, quercetin^82^,5,7-dihydroxy-2',6-dimethoxyisoflavone^83^,tectorigenin^84^ and genistein ^85^. In the same context, IPR-J activity could be attributed to vitexin^6^, isovitexin^6^, quercetin^82^ and apigenin-*O*-hexoside^86^. All these constituents act in synergistic mechanism with the other phenolic compounds found in IPR-E and IPR-J. Quercetin was recognized as an *α*-glucosidase inhibitor similar to acarbose. It was reported to delay glucose absorption and prevent the digestion of carbohydrates by inhibiting the sucrase, maltase and *α*-amylase. It also promotes proliferation of pancreatic beta cell, thus, improving absorption of glucose^87^. 5, 7-Dihydroxy-2', 6-dimethoxyisoflavone was reported as an effective anti-diabetic agent. It exhibited excellent activity against *α*-glucosidase enzyme with IC_50_ value at 0.321±0.008 µg/mL compared to acarbose IC_50_ value at 1.52±0.004 µg/mL at the same time it inhibited protein glycation strongly with % inhibition of 70.41 at concentration of 3 µg/mL as compared to rutin (82.50%). Noteworthy, inhibition of protein glycation lead to delay of diabetic complications as neuropathy and nephropathy^83^. An in vivo study on normoglycemic and induced diabetic rats examined the antihyperglycemic activity of vitexin and isovitexin. It was found that the highest reduction in postprandial blood glucose level was in induced diabetic rats treated orally with 200 mg/kg of vitexin and 100 mg/kg of isovitexin and the percentage of the reduction was similar to acarbose. Vitexin and isovitexin displayed strong in vitro α-glucosidase inhibition with IC_50_ values of 4.1 and 6.7 µg/mL compared with acarbose IC_50_ value at 4.3 × 10^-2^ µg/mL^6^. Genistein was reported as a potent *α*-glucosidase inhibitor, it remarkably inhibited *α*-glucosidase enzyme with IC_50_ values of 40.09 ± 0.94 µg/mL compared with acarbose IC_50_ value at 296.6 ± 1.06 µg/mL ^85^. To the best of our knowledge, the present study is the first regarding the anti-hyperglycemic activity of *I. pseudacorus*. At this point, the presented results could open novel perspectives for designing new plant-based nutraceuticals.

The inhibition of pancreatic lipase is considered a precious approach for the management of diet-induced hyperglycemia (one of causes of diabetes mellitus) and obesity. The effectiveness of *I. pseudacorus* extracts as a promising anti-hyperlipidemic could be attributed to its richness in flavonoids. These secondary metabolites were previously showed to possess anti-hyperlipidaemic activity *via* acting as potent pancreatic lipase inhibitors. Noteworthy, few studies evaluated the pancreatic lipase inhibitory activity of various *Iris* species. Several phytoconstituents identified herein in *I. pseudacorus* extracts were previously reported as natural pancreatic lipase inhibitory compounds including schaftoside ^88^, orientin ^89^, isovitexin ^89^, kaempferol-3-O-rutinoside^90^, quercetin^91^, genistein^92^. Pancreatic lipase enzyme displayed a binding pocket for quercetin. Once quercetin was attached with the lipase resulted in conformation changes lessening the substrate - enzyme affinity. Quercetin pre-administration in rats (5 and 10 mg/kg) through in vivo studies lead to a remarkable decrease in rat fat absorption and increase its excretion. Quercetin strongly inhibited pancreatic lipase enzyme through in vitro studies with IC_50_ value at 70 μg/mL compared with orlistat IC_50_ 80 μg/mL^91^. Schaftoside exhibited a potent pancreatic lipase inhibitory activity with % inhibition of 95.5% at a concentration of 250 μg/mL and IC_50_ value of 130 μg/mL compared with orlistat IC_50_ value at 98.80 μg/mL ^88^. Kaempferol-3-*O*-rutinoside showed effective pancreatic lipase inhibitory activity with IC_50_ value of 1.7± 0.30 μg/mL compared to orlistat IC_50_ 0.72 ± 0.07 μg/mL^90^.

*In silico* molecular docking studies were performed to further confirm the obtained in vitro results and identify the possible interaction mechanisms between *I. pseudacorus* phytoconstituents within the active sites of human *α*-glucosidase (HAG) and human pancreatic lipase (HPL). The firm fitting between the hit compounds and the enzyme active sites could be attributed to the formation of several bonds including π-π bond, H-bond, C-H bond and Van der Waals forces with the amino acid moieties in the enzyme binding site. Besides, ADMET prediction was conducted to examine whether *I. pseudacorus* phytoconstituents possess drug-like properties or not. It is an essential step in the pharmaceutical R&D development.

## Materials and methods

### Chemicals and reagents

2, 2-Diphenyl-1-picrylhydrazyl (DPPH), pancreatic lipase enzyme from porcine animal, tyrosinase enzyme from mushroom plant and *α*-glucosidase enzyme from *Saccharomyces cerevisiae* fungi were purchased from the Sigma Aldrich, Japan. Arbutin and aluminum chloride (III) were obtained from Nacalai Tesque, Japan. Cetilistat was purchased from Combi-Blocks, United States of America. Acarbose and Trolox were obtained from Wako Pure Chemical Industries, Japan. Methanol was purchased from Al-Nasr Pharmaceutical Company, Egypt.

### Plant material

Aerial parts and rhizomes of *I. pseudacorus* were collected in January 2019 from El-Orman botanical garden, Giza, Egypt (30°01′45″ N 31°12′47″ E). The plant was identified and authenticated morphologically by Eng. Therease Labib, consultant of plant taxonomy at the Ministry of Agriculture, National Gene Bank and El-Orman Botanical Garden, Egypt. The collection complied with the IUCN Policy Statement on Research Involving Species at Risk of extinction and collection requirements were carefully followed in the conduct of this research to comply with institutional, national, and international guidelines and legislation. A voucher specimen (No. PHG-P-IP-417) was deposited in the Herbarium of Pharmacognosy Department, Faculty of Pharmacy, Ain Shams University. Aerial parts and rhizomes of *I. pseudacorus* cultivated in Japan were collected from the Medicinal Plant Garden of Kumamoto University, Japan (N32.794649, E130.72206), in December 2018, and were authenticated by Mr. Masato Watanabe, School of Pharmacy, Kumamoto University in Japan. A voucher specimen (No. 20181201-001) was deposited in the Herbarium of Medicinal Plant Garden of Kumamoto University. The metabolic profiling of aerial parts and rhizomes of Egyptian and Japanese *I. pseudacorus* extracts was carried out at the Center for Drug Discovery Research and Development, Faculty of Pharmacy, Ain Shams University, Cairo, Egypt. Meanwhile, in vitro assays were performed at the school of pharmacy, Kumamoto University in Japan.

### Preparation of plant extracts

The dried aerial parts and rhizomes of *I. pseudacorus* cultivars were air-dried in the shade, cut into small pieces. Egyptian and Japanese aerial parts of *I. pseudacorus,* 57 g and 11.1 g, respectively, besides, 69 g of Egyptian rhizomes and 10 g of Japanese rhizomes were extracted twice with 100% methanol (3L x 2) using a sonicator bath (690 HTAE Crest) for 60 minutes each time. Extracts were filtered by cotton fabric filter media. Extracts were concentrated using rotatory vacuum evaporator (Büchi, Switzerland) under reduced pressure at 45 °C and completely dried using a lyophilizer (Christ, Alpha 1–2 LD Plus) to yield 6 g, 1.16 g, 3 g and 0.43 g dried extracts of IPA-E, IPA-J, IPR-E and IPR-J, respectively.

### Phytochemical analysis

#### UPLC-ESI-MS/MS characterization of *I. pseudacorus* extracts

Metabolic profiling of aerial parts and rhizomes of Egyptian and Japanese *I. pseudacorus* extracts was carried out on a Waters Xevo TQD mass spectrometer with UPLC Acquity mode (Milford, CT, USA) at the Center for Drug Discovery Research and Development, Faculty of Pharmacy, Ain Shams University. Extracts were liquefied in diluted methanol and injected directly into the UPLC-ESI-MS system. Both negative and positive ESI ionization modes were applied under the following conditions: A gradient of water and acetonitrile (ACN) with 0.1% was applied from 2 to 100% ACN in 60 min at 30 ◦C. The flow rate was 0.5 mL/min. The injection volume was 20 µL. The capillary voltage of MS (10 V), the ions were noticed within a mass range from 50 to 2000 *m*/*z* with collision energy (35 eV).

### In Vitro biological evaluation

#### In Vitro evaluation of antioxidant activity using DPPH assay

The antioxidant activity of the yellow flag extracts was determined using DPPH scavenging assay in accordance with Shimamura et al. with minimal adjustments ^93^. The reaction mixture in 96 well culture plate contained. *I. pseudacorus* extracts (25 μL with several concentration ranges till 125 µg/mL), MES buffer (50 μL as volume and concentration =200 mM at pH = 6.0), 50% diluted ethanol (75 μL) mixed with 2, 2-diphenyl-1-picrylhydrazyl solution in EtOH (50 μL, 800 μM). After 20 min of incubation of the reaction mixture at 25 °C. The degree of bleaching of the violet tint of DPPH depends on the ability of *I. pseudacorus* extracts for hydrogen/electron donation and was measured spectrophotometrically at 520 nm of wavelength. Noteworthy, trolox (an artificial anti-oxidant agent) was used as a positive control. The percentage of antioxidant capacity of the yellow flag extracts equals to [$\frac{Ac-As}{Ac}$] *100 Where Ac, As represents the control absorbance and the *I. pseudacorus* extract absorbance, respectively. The graph was plotted and the inhibitory concentration 50 (IC_50_) value was calculated^93^.

#### Enzyme inhibitory activity

##### In Vitro anti-hyperglycemic activity using *α*-glucosidase enzyme assay

The anti-hyperglycemic potential of the yellow flag extracts was conducted in accordance with Jabeen et al. method with slight alterations ^93^. The reaction mixture in 96 well culture plate contained a solution of *α*-glucosidase enzyme dissolved in phosphate buffer (10 μL, 1 UN/mL), 10 μL of the tested *I. pseudacorus* extract. Noteworthy, the range of extracts concentration for the *α*-glucosidase inhibition assay was 1.56 to 100 μg/mL mixed with phosphate buffer (60 μL, 0.2 M at pH 6.8). The mixture was incubated at 37 °C for 5 min. Subsequently, *p*-nitrophenyl *α*-D-glucoside (20 μL, 4 mM) was added to the reaction mixture as a substrate. Then, the reaction mixture was kept warm for 12 min at 37 °C. The amount of *p*-nitrophenol liberated by *α*-glucosidase enzyme was measured spectrophotometrically at 405 nm. The percentage of *α*-glucosidase inhibition potential of yellow flag extract was evaluated using the following equation: [1 – $\frac{\mathrm{Aa }-\mathrm{ Ab}}{\mathrm{Ac }-\mathrm{ Ad}}$]*100, in which Aa, Ab, Ac and Ad signify *α*-glucosidase and tested extracts absorbance, tested extracts absorbance only, *α*-glucosidase absorbance only, absorbance in absence of both, respectively. Acarbose (an *α*-glucosidase inhibitor) was used as a positive control. The graph was plotted and the concentration required to inhibit half-life of *α*-glucosidase function (IC_50_) was obtained ^93^.

##### In Vitro anti- hyperlipidaemic activity using pancreatic lipase enzyme assay

The anti-hyperlipidemic potential of yellow flag extracts was conducted by Bitou et al. method with minor alterations^93^. In an ELISA microplate reader, pancreatic lipase solution (50 μL at pH 7.4) in phosphate buffer (100 μg/mL, 0.2 M) was added together with 50 μL of *I. pseudacorus* extract at a concentration ranging from 1.56 to 100 μg/mL. After 10 min of incubation at 25 °C. 4-methylumbelliferyl oleate (4MUFO) (100 μL, 0.5 mM) was added to the reaction mixture as a substrate. The quantity of 4-methylumbelliferone liberated by the lipase enzyme was measured fluorometrically using a spectrophotometer at 355 nm excitation wavelength and 460 nm emission wavelength. Cetilistat (an artificial lipase inhibitor) was used as a positive control. The percentage of lipase inhibition was calculated using the following equation: [1- $$\frac{\begin{array}{c}As\\ \end{array}}{\mathrm{Ac}}$$ ]*100], where As and Ac symbolize *I. pseudacorus* extract and cetilistat absorbances. The graph was plotted and the concentration required to inhibit half-life of lipase activity (IC_50_) was obtained^93^.

##### In Vitro anti-melanogenesis activity using tyrosinase enzyme assay

The anti-melanogenesis potential of the yellow flag extracts was evaluated in accordance with Adhikari et al. method with minor changes^93^. The reaction mixture in 96 well culture plate contained phosphate buffer (120 μL at pH 6.8), tyrosinase solution in phosphate buffer (50 μL, 100 u/mL)) and 10 μL of *I. pseudacorus* extract. After 10 min of incubation at 25 °C, 20 μL of 2 mM of L-tyrosine substrate was added. The spectrophotometric absorbance was acquired at 476 nm after two and ten min. The percentage of tyrosinase inhibition was determined employing the following equation, [1- $$\frac{\begin{array}{c}As\\ \end{array}}{\mathrm{Ac}}$$ ]* 100, where As represents the absorbance difference of *I. pseudacorus* extract at the incubation time ten and two min and Ac denotes the absorbance difference of arbutin at the incubation time of ten and two min. Arbutin (a tyrosinase inhibitor) was used as a positive control. The graph was plotted and the concentration required to inhibit half-life of tyrosinase function (IC_50_) was obtained^93^.

### Molecular docking studies

#### Molecular docking

*In silico* molecular docking study was performed on the major compounds identified in *Iris pseudacorus* extracts to elucidate the putative binding mode to the active sites of *α*-glucosidase and pancreatic lipase in an attempt to predict their probable mode of action as anti-hyperglycemic and anti-hyperlipedemic. Crystal structures of human α-glucosidase (HAG) (PDB ID 3TOP; 2.88 Å) and human pancreatic lipase (HPL) (PDB ID 1LPB; 2.46 Å) were retrieved from the protein data bank (www.pdb.org, accessed on 24 September 2022). Discovery Studio 4.5 (Accelrys Inc., San Diego, CA, USA) was employed applying the C-docker protocol as previously described ^94^.

#### ADMET prediction

Absorption, distribution, metabolism, excretion, and toxicity (ADMET) were predicted for the major metabolites identified in *I. pseudacorus* extracts employing ADMET prediction protocol using Discovery Studio 4.5 (Accelrys Inc., San Diego, CA, USA).

### Statistical analysis

DPPH and enzyme inhibitory assays were carried out in triplicates, and the values were expressed as mean *±* standard deviation. For the determination of the in vitro antioxidant, tyrosinase, lipase, and *α*-glucosidase inhibition potential, the (IC_50_) was estimated from the graph plots of the dose–response curves at each sample concentration by GraphPad Prism software (San Diego, CA, USA). The IC_50_ was defined as the concentration of the sample required to inhibit 50% of the tested enzyme activity.

## Conclusions

The current study investigated for the first time the phytochemical diversity of *I. pseudacorus* aerial parts and rhizomes from Egypt and Japan. Besides, their potential inhibitory activity on selected enzymes (*α*-glucosidase, lipase and tyrosinase) were evaluated for the first time. Furthermore, their antioxidant capacities were assessed. Additionally, *in Silico* studies were performed to identify the possible interaction mechanisms between *I. pseudacorus* phytoconstituents and their targets to further validate the obtained in vitro results. Furthermore, ADMET prediction were conducted to evaluate their pharmacokinetics, pharmacodynamics and toxicity properties. Iris metabolites profiling was performed using UPLC-ESI-MS/MS analysis in an attempt to correlate the identified metabolites with the observed activities. Metabolites profiling revealed richness of *I. pseudacorus* extracts with biologically active compounds including schaftoside, orientin, isovitexin, tectorigenin, genistein, irilin D, quercetin and irisolidone. The rhizome methanol extracts of Egyptian and Japanese *I. pseudacorus* showed significant antioxidant activity and antihyperglycemic activity. Moreover, all investigated *I. pseudacorus* extracts showed potent anti-hyperlipidaemic activity *via* lipase enzyme inhibition. Results revealed that the rhizomes methanol extract of Japanese *I. pseudacorus* has the highest antioxidant, antihyperglycemic and anti-hyperlipidaemic activity among the other investigated extracts. Additionally, *in silico* molecular docking revealed that quercetin, galloyl glucose, and irilin D exhibited the highest fitting scores within the active sites of human* α*-glucosidase and pancreatic lipase. Moreover, most of phytoconstituents displayed promising pharmacokinetics, good pharmacodynamics and tolerable toxicity properties in ADMET plot. Consequently, the current study revealed that Egyptian and Japanese *I. pseudacorus* rhizomes may be considered as a promising natural source of antioxidant and antidiabetic agents. Furthermore, *I. pseudacorus* rhizomes and aerial parts of each cultivar should be taken into consideration as a valuable source for innovating new phytopharmaceuticals in the field of obesity as antihyperlipidaemic drugs. It is highly recommended to conduct further research for isolation of pure compounds from Egyptian and Japanese *I. pseudacorus* to explore the putative underlying mechanisms for the observed antihyperglycemic and antihyperlipidaemic activities. Additionally, further research should be conducted for elucidating in vivo enzyme inhibition activity besides, in depth studies are needed to confirm their efficacy and safety.

## Data Availability

Data are available upon request from the first author, Suzan M. Yehia.
